# Penalized Regression Methods With Modified Cross‐Validation and Bootstrap Tuning Produce Better Prediction Models

**DOI:** 10.1002/bimj.202300245

**Published:** 2024-06-24

**Authors:** Menelaos Pavlou, Rumana Z. Omar, Gareth Ambler

**Affiliations:** ^1^ Department of Statistical Science UCL London UK

## Abstract

Risk prediction models fitted using maximum likelihood estimation (MLE) are often overfitted resulting in predictions that are too extreme and a calibration slope (CS) less than 1. Penalized methods, such as Ridge and Lasso, have been suggested as a solution to this problem as they tend to shrink regression coefficients toward zero, resulting in predictions closer to the average. The amount of shrinkage is regulated by a tuning parameter, λ, commonly selected via cross‐validation (“standard tuning”). Though penalized methods have been found to improve calibration on average, they often over‐shrink and exhibit large variability in the selected λ and hence the CS. This is a problem, particularly for small sample sizes, but also when using sample sizes recommended to control overfitting. We consider whether these problems are partly due to selecting λ using cross‐validation with “training” datasets of reduced size compared to the original development sample, resulting in an over‐estimation of λ and, hence, excessive shrinkage. We propose a modified cross‐validation tuning method (“modified tuning”), which estimates λ from a pseudo‐development dataset obtained via bootstrapping from the original dataset, albeit of larger size, such that the resulting cross‐validation training datasets are of the same size as the original dataset. Modified tuning can be easily implemented in standard software and is closely related to bootstrap selection of the tuning parameter (“bootstrap tuning”). We evaluated modified and bootstrap tuning for Ridge and Lasso in simulated and real data using recommended sample sizes, and sizes slightly lower and higher. They substantially improved the selection of λ, resulting in improved CS compared to the standard tuning method. They also improved predictions compared to MLE.

## 1 Introduction

Risk prediction models are routinely used in clinical practice to assist medical decision‐making. These are often derived by fitting a regression model to the available data (“development data”). For binary outcomes, logistic regression is the usual choice. The performance of a risk model must be evaluated in an external dataset (“validation dataset”) before the model can be used in practice to make predictions for new patients. This evaluation may include the calculation of measures of calibration (calibration slope [CS] and calibration in‐the‐large), discrimination (*C*‐statistic), overall predictive accuracy (Brier Score), and net benefit.

When the sample size is small relative to the number of regression parameters, the resulting logistic regression model fitted using maximum likelihood estimation (MLE) tends to be overfitted, that is, tends to provide predictions for new patients that are too extreme. A CS ≪ 1 on external data is indicative of model overfitting (Steyerberg et al. 2010). In such cases, penalized logistic regression methods such as Ridge and Lasso regression tend to shrink the regression coefficients toward zero, and this can be beneficial in reducing model overfitting. The amount of shrinkage is regulated by a shrinkage parameter, which is typically estimated using cross‐validation to maximize the out‐of‐sample performance of the model.

Several studies have investigated the performance of penalized regression in low‐dimensional settings using simulation (Pavlou et al. 2016; Steyerberg, Eijkemans, and Habbema 2001; Steyerberg et al. 2000; van Houwelingen 2001). Recently, Van Calster et al. (2020) and Šinkovec et al. (2021) used simulation to evaluate the performance of penalized methods for a range of events‐per‐variable scenarios (2.5–50) with varying outcome prevalence, number of (noise and true) predictors, and correlation between predictors. Riley et al. (2021) performed similar investigations with higher prevalence (0.5) when the true *C*‐statistic was 0.7. Penalized methods were found to be effective in reducing model overfitting on average (Pavlou et al. 2016). In some cases, they were seen to over‐shrink, as indicated by an average CS > 1. Importantly, they exhibited large variability in the estimation of the tuning parameters and, consequently, of the CS. When combining bias and variability of the log(CS) in a single‐performance measure such as the “root mean square distance” from the target value (RMSD), penalized methods often performed worse than MLE. The uncertainty in the degree of shrinkage and the large variability of the CS were interpreted to mean that penalized methods tend to perform well on average but may perform poorly in individual datasets. We note that for small datasets, separation may occur, in which case MLE may not have a solution, whereas penalized methods tend to apply minimal or no shrinkage, hence not being helpful in dealing either with separation or model overfitting (Šinkovec et al. 2021). For those occasions, methods that specifically handle separation (Firth 1993) are recommended.

Recently, Riley et al. (2019) proposed sample size calculations to control model overfitting and other measures of predictive performance. Martin et al. (2021) evaluated the performance of penalized methods when these sample size recommendations were met or surpassed. Even though in the settings investigated, the sample sizes were relatively large, they still found that, due to the large variability in the CS, the RMSD of the CS for penalized methods was usually higher or equal to that for the MLE. This was interpreted as a high chance of resulting in a miscalibrated model in external validation when penalized methods are used.

In this paper, we consider relatively large datasets, with sample sizes close to the recommended. We propose a modified cross‐validation tuning method (hereafter referred to as “modified tuning”), which can be easily implemented in standard software. The modified tuning method is closely related to bootstrap selection of the tuning parameters (hereafter referred to as “bootstrap tuning”), which has been rarely used in practice. We explore whether the modified and bootstrap tuning methods can be used to reduce the variability in the selected tuning parameter and consequently improve the performance of penalized methods in terms of calibration. We consider binary outcomes. In Section 2, we describe standard penalized regression methods, and in Section 3, we present the modified and bootstrap tuning approaches that are suitable for penalized methods that require tuning (e.g., Ridge and Lasso). In Section 4, we consider sample sizes close to the recommended, and we use simulation to compare the predictive performance of models developed by penalized methods with either modified tuning or bootstrap tuning to the performance of models developed using penalized methods with standard tuning, MLE, uniform shrinkage, and Firth's (1993) method. In Section 5, we apply the methods to a real cardiac dataset. We conclude with a discussion regarding the usefulness of the modified tuning and bootstrap tuning methods in practice and caveats regarding their use.

## 2 Risk Prediction, Uniform Shrinkage Methods, and Penalized Regression

Let $Y_{i}$ denote the binary outcome and $x_{i}={\left(1,x_{i1},x_{i2},\text{…},x_{ip}\right)}^{T}$ a (*p* + 1)‐dimensional vector of covariate values for the *i*‐th individual, i=1,…n. The probability of experiencing the event of interest is typically modeled using a logistic regression model for $P(Y_{i}=1)$:

(1) $$\mathrm{logit}\left(P\left(Y_{i}\right)=1\right)=\beta^{T}\;x_{i}$$

where $\beta ={\left(\beta_{0},\beta_{1},\text{…}\beta_{p}\right)}^{T}$ is a vector of regression coefficients. Typically, β is estimated using MLE. The dataset used to fit the model is called development or training dataset. For a given observation, the estimated probability of having the event of interest is computed by ${\hat{\pi }}_{i}={\mathrm{logit}}^{-1}$(${\hat{\beta }}^{T}x_{i}$) where $\hat{\beta }$ is the MLE estimate of β.

The estimated regression coefficients can be used to obtain predictions for new patients, based on their patient characteristics. However, before a risk model can be used in clinical practice, its predictive performance needs to be assessed in new data (validation or test data) that are different from the development data. Metrics considered for the validation of a risk model often address calibration, discrimination, and overall predictive accuracy.

Calibration refers to the agreement between observed and predicted values in an overall sense (calibration‐in‐the large) and for different risk groups (CS) (Van Calster et al. 2016). CS is the slope term in a logistic regression model, where the linear predictor is regressed on the binary outcome. For the calibration in‐the‐large, a similar model as above is fitted, but the linear predictor included is an offset term (with slope equal to one). The intercept term in this model is the calibration in the large. A value of 0 for the calibration in‐the‐large suggests that the average predicted probability is equal to the observed proportion of events. A value of 1 for the CS suggests a perfectly calibrated model. The *C*‐statistic is a measure of model discrimination. Considering two patients, one with and one without the event, the *C*‐statistic is the probability that the patient with the event is assigned the higher predicted risk. It takes values between 0.5 and 1, with higher values meaning higher discriminatory ability. Overall predictive accuracy can be assessed with the Brier Score (the average of the squared differences between the outcome and the estimated probabilities). A lower Brier Score suggests a more accurate model.

When the sample size is small relative to the number of regression parameters, the model tends to be fitted too well on the development data. As a result, the estimated coefficients are typically too large in magnitude, and consequently, high predictions are too high and low predictions too low. This is known as model overfitting. A value for the CS < 1 is suggestive of model overfitting (Miller, Hui, and Tierney 1991). Applying some shrinkage to the estimated values of the regression coefficients may help in reducing model overfitting. In the next sessions, we will briefly describe two categories of shrinkage methods. Further detail on these and other methods can be found elsewhere (Martin et al. 2021; Pavlou et al. 2016; Van Calster et al. 2020).

### 2.1 Uniform Shrinkage Methods

In practice, shrinkage is often used to improve the predictions from a logistic regression model (van Houwelingen 2001). This first category of shrinkage methods applies a (uniform) shrinkage factor to the coefficient estimates after the model has been fitted using MLE. This has the effect of shrinking all MLE coefficient estimates toward zero, which shrinks the corresponding predictions toward the overall outcome prevalence.

The shrinkage factor is most often estimated using the bootstrap (Efron and Gong 1983). Briefly, the model is fitted to bootstrap datasets, with the original dataset used for validation. The average value of the CS over these bootstrap datasets is an estimate of the shrinkage factor (Efron and Gong 1983). After shrinkage has been applied, the intercept term is re‐estimated so that the average of the predicted probabilities equals the outcome prevalence.

### 2.2 Penalized Regression Methods

Penalized methods impose restrictions on the values of regression coefficients, which result in the shrinkage of the estimated coefficients as part of the estimation process. This is achieved by maximizing a “penalized” log‐likelihood function, which takes the form:

$$l\left(\beta \right)-\lambda s\left(\beta \right),$$

where l(β) is the log‐likelihood function for model (1), s(β) is a penalty term that can take different functional forms resulting in different shrinkage patterns, and λ is a tuning parameter that regulates the amount of shrinkage.

In terms of penalized methods that require tuning, that is, choosing the tuning parameter λ, we focus on two commonly used penalty terms for s(β): $\sum_{j=1}^{p}\beta_{j}^{2}$, which corresponds to Ridge regression (L2 penalization) (Cessie and Houwelingen 1992), and $s\left(\beta \right)=\sum_{j=1}^{p}\left|\beta_{j}\right|$, which corresponds to Lasso regression (L1 penalization) (Tibshirani 1994). Lasso regression may shrink some of the coefficients to exactly zero, thus performing variable selection. We focus on Ridge and Lasso regression since they are more suited to low‐dimensional settings than other methods (e.g., adaptive Lasso) (Pavlou et al. 2016).

The tuning parameter λ is typically obtained using cross‐validation to maximize the out‐of‐sample predictive performance of the model. The choice of λ is a crucial aspect of fitting a model using penalized methods. A value of λ that is too small will result in insufficient shrinkage (λ=0 corresponds to MLE), whereas a value of λ that is too large will result in excessive shrinkage and model underfitting, as indicated by a CS > 1 in validation data, suggesting that the range of predictions is too narrow.

In practice and in most of the previous method evaluations, 5‐ or 10‐fold cross‐validation is used for tuning (10‐fold is the default option in many software packages, and this number of folds is primarily used in this paper).

Firth's bias reduction method (Firth 1993; Heinze and Schemper 2002) is also a penalized method, and it was originally proposed to solve separation problems in logistic regression. The penalized likelihood for Firth's method is

$$l\left(\beta \right)+0.5\log\left|I\left(\beta \right)\right|$$

where I(β) is Fisher's information matrix. Contrary to Ridge and Lasso, Firth's method does not involve a tuning parameter. As the method also shrinks coefficients toward zero, it has been recently considered, with intercept correction that avoids biasing the average predicted probability toward 0.5, alongside other shrinkage methods to reduce model overfitting (Martin et al. 2021; Šinkovec et al. 2021; Van Calster et al. 2020).

## 3 Improved Tuning for Penalized Methods Used for Prediction

Typically, the tuning parameter for penalized regression methods is chosen to maximize the out‐of‐sample performance of the model. Most commonly, this is done using cross‐validation, which is described below.

### 3.1 Standard Tuning Algorithm With *k*‐Fold Cross‐Validation

**Step 1**: The (original) development dataset of size n is randomly split in k parts. Commonly used values are k=5 or 10.
**Step 2**: (k−1) parts form a cross‐validation training dataset (cv‐training set) and the left‐out part serves as a cross‐validation test dataset (cv‐test set). A sequence of values for λ is chosen, and for each of those values, the model is fitted on the cv‐training set using the penalized method of choice.
**Step 3**: Step 2 is repeated *k*‐times, each time leaving out a different part.
**Step 4**: A measure of out‐of‐sample performance (such as deviance, *C*‐statistic, mean absolute error) is calculated using the cv‐test sets for each value of *λ*. Here, we consider the deviance.
**Step 5**: The selected value of $\lambda ,\lambda_{\min}$, is the one that minimizes the cross‐validated deviance.
**Step 6**: The regression coefficients are estimated by fitting the model using the penalized method of choice in the *original development dataset* with $\lambda =\lambda_{\min}$.

Steps 1–6 are performed in standard software, with k=10 usually being the default value. In this paper, we fit the penalized methods using the R package “glmnet.”

With respect to the algorithm above, we note two issues. First, depending on the random split of the data in Step 1, a slightly different optimal value of λ will be chosen. To decrease the uncertainty in the choice of λ and to avoid extreme values of λ due to an unfortunate split, repeated cross‐validation can be used and the median value of λ be selected. In previous studies (Pavlou et al. 2016; Šinkovec et al. 2021), this, however, was seen to have relatively minor effect in reducing uncertainty in the estimation of λ and the CS.

Second, the optimal value of λ is obtained using cv‐training sets in Steps 2 and 3 that are *smaller* than the original development dataset. In particular, the size of each of the cv‐training datasets is $n\times \frac{k-1}{k}$. For example, when k=10, the size of each cv‐training set is 10% smaller than the original dataset. When dealing with small development datasets or datasets with very few events, this reduction in size is non‐negligible when it comes to optimizing the tuning parameter in Step 2. As tuning is performed on a smaller dataset than the original, the optimal λ tends to be overestimated, which may contribute to excessive shrinkage and uncertainty in the estimation of λ for some of the penalized methods. The effect will be more pronounced for a smaller number of cross‐validation folds. On the other hand, for very large datasets, the impact of reduction in the size of the cv‐training sets should be less pronounced (Steyerberg et al. 2001).

These observations are in line with the results of previous simulation studies. For instance, when five‐fold cross‐validation was used previously (Riley et al. 2021) and the sample size was very small, the effect of over‐shrinkage, and increased variability was particularly pronounced. As part of sensitivity analysis in the simulation study of Section 4, we study the effect of the number of cross‐validation folds.

We hypothesize that obtaining the optimal value of λ on cv‐training sets of the same size as the original dataset will improve the estimation of λ. To obtain such cv‐training sets, we propose starting with a larger pseudo‐dataset by sampling with replacement from the original dataset, such that in Step 2 the cv‐training sets are of the same size as the original development dataset. We propose the following modified tuning algorithm.

### 3.2 Modified Tuning With *k*‐Fold Cross‐Validation (“Modified Tuning”)

**Step 0**: Create a sample from the original development by sampling with replacement to create a pseudo‐dataset of size $n_{\mathrm{pseudo}}=n\times \frac{k}{k-1}$, that is, *larger* than the original dataset.
**Steps 1–4** are the same as for the standard tuning algorithm, but starting with the pseudo‐development dataset of Step 0. Importantly, the resulting cv‐training sets in *k*‐fold cross‐validation are now of the *same size as the original dataset*.
**Step 5**: Repeat Steps 0–4 *B* times and choose the value of λ, $\lambda_{\mathrm{mod}}$, that minimizes the averaged cross‐validated deviance over the *B* iterations (alternatively, first obtain $\lambda_{\min}$ in each of the *B* datasets and then choose the median value as $\lambda_{\mathrm{mod}}$, which gives effectively identical results).
**Step 6**: Fit the model to the original dataset using the chosen penalized method with the chosen value of the tuning parameter, $\lambda_{\mathrm{mod}}$.

Note that Steps 1–4 are computed using standard software, and so the modified tuning algorithm only requires accommodating Steps 0, 5, and 6. Step 0 serves the purpose of avoiding excessive shrinkage, whereas Step 5 further increases stability in the selection of λ. We suggest *B* = 100, but even with *B* as low as 50, results were almost identical to *B* = 100 or higher (results not shown). We also note that the method can be applied in the same way for time‐to‐event data.

### 3.3 Bootstrap Tuning

An alternative, closely related approach for tuning is the standard bootstrap method, where for each value (in a sequence of values) of λ, the model is fitted on a bootstrap sample and the out‐of‐sample performance is assessed on the original sample. We call this approach “Bootstrap tuning.” The chosen value of λ is the one that optimizes the average of an out‐of‐sample performance measure (e.g., deviance) over repeated bootstrap samples.

The modified and bootstrap tuning methods are closely related. Effectively, the modified cross‐validation approach uses bootstrap samples (of size n) to fit the models and bootstrap samples (of size n/10) to validate them. Hence, the two approaches are expected to perform similarly, although modified tuning is arguably more straightforward to apply in practice, because usually software for penalized regression uses cross‐validation by default. On the other hand, the bootstrap approach requires the user to manually calculate the out‐of‐sample performance measure to be maximized on the original validation dataset after fitting the model on the bootstrap samples for all values of λ. Here we focus on the standard bootstrap for tuning; variations such as the 0.632 and 0.632+ bootstrap may also be used (Efron and Tibshirani 1997), although in the related context of internal validation of prediction models, they were not found to be superior to the standard bootstrap (Steyerberg et al. 2001). The modified and bootstrap tuning methods are compared later in the simulation study.

## 4 Simulation Study

### 4.1 Simulation Setup

#### 4.1.1 Aims

We aim to investigate the predictive performance of models developed using penalized methods with modified and bootstrap tuning in comparison to using penalized methods with standard tuning and MLE. We use performance measures that combine the bias and variability of the CS. We investigate the performance of the methods close to the sample sizes recommended to limit model overfitting (target expected shrinkage of 0.9).

#### 4.1.2 Methods

The estimation methods we use are maximum likelihood (“MLE”), Firth's logistic regression with intercept correction (“Firth”), bootstrap linear shrinkage factor (“Boot‐Unif”) and Ridge and Lasso regression with standard, modified, and bootstrap tuning (“Ridge,” “Lasso,” “Mod‐Ridge,” “Mod‐Lasso,” “Boot‐Ridge,” and “Boot‐Lasso”). Modified tuning and bootstrap tuning are applied with *B* = 100 samples and 10‐fold cross‐validation. In line with previous studies that investigated the performance of penalized methods, standard tuning is performed using 10‐fold cross‐validation. For a subset of scenarios, the effect of the number of cross‐validation folds on the standard tuning method is determined by considering 5, 10, and 20‐fold and leave‐one‐out (*n*‐fold) cross‐validation.

#### 4.1.3 Data‐Generating Mechanism

For each scenario, we generate binary outcomes from a logistic regression model. We consider p predictor variables from a multivariate normal distribution with mean zero and correlation matrix Σ. We let x denote the vector of covariate values for a given observation. Some of these predictors are “true” predictors with their corresponding regression coefficients being non‐zero, and some are “noise” predictors with their corresponding coefficients being zero. The linear predictor is $\eta_{i}=\beta^{T}x_{i},i=1,\text{…}n$ (n is the sample size). The binary outcome is generated from a logistic regression model, that is, $y_{i}∼\mathrm{Bernoulli}\;\left(\pi_{i}\right),$ where $\pi_{i}={\mathrm{logit}}^{-1}\;\left(\eta_{i}\right)$ are the true predicted probabilities. The regression coefficients $\beta_{1},\text{…},\beta_{p}$ are chosen to reflect the strength of the model (as reflected by the *C*‐statistic), and the intercept term, $\beta_{0}$ is chosen to set the outcome prevalence.

We generate large validation datasets of size $n_{\mathrm{val}}=50,000$ from the data‐generating mechanism above. The development datasets are generated using the same data‐generating mechanism. For each scenario, we generate $n_{\mathrm{sim}}=1000$ development and validation datasets. We then fit models to each of the development datasets using each of the fitting methods and calculate measures of predictive performance (see below) using the validation datasets.

#### 4.1.4 Performance Measures

We evaluate the performance of the methods on average in terms of calibration (CS), discrimination (*C*‐statistic), and overall predictive accuracy (root mean square prediction error (RMSPE), which is the square root of the average of the squared differences between the true and the estimated probabilities). For the average performance of the methods, we calculate the median of the estimated measure of predictive performance over the $n_{\mathrm{sim}}$ values. For CS, the optimal value is 1.

We then focus on the CS and compare the methods in terms of measures that combine bias and variance of the CS. Following Van Calster et al. (2020), we calculate the root mean square distance (RMSD) from the target value of the log of the CS:

$$\mathrm{RMSD}(\log-\mathrm{CS})=\sqrt{\frac{1}{n_{\mathrm{sim}}}\sum_{i=1}^{n_{\mathrm{sim}}}{\left(\log\left({\mathrm{CS}}_{i}\right)-\log\left(\mathrm{target}\right)\right)}^{2}}$$

where ${\mathrm{CS}}_{\hat{i}}$ is the CS in the *i*‐th dataset, and the target value of the CS is 1, corresponding to a perfectly calibrated model. The method that leads to the lowest RMSD(log − CS) is taken to be the best performing method for a given scenario.

We also compare the methods in terms of the probability of providing a “well‐calibrated” model. We define a well‐calibrated model as one with CS between 0.9 and 1.1 in external data. This probability is approximated by the proportion of times over the $n_{\mathrm{sim}}$ simulations that the CS is between 0.9 and 1.1:

$$p_{\mathrm{well}\_\mathrm{cal}}=\frac{1}{n_{\mathrm{sim}}}\;\sum_{i=1}^{n_{\mathrm{sim}}}I\left({\mathrm{CS}}_{i}\geq 0.9\;\&\;{\mathrm{CS}}_{i}\leq 1.1\right)$$

#### 4.1.5 Simulation Scenarios and Parameter Values

For our main simulation, we generate data with p=12 predictors, 5 of which are true and 7 are noise. We write the vector of regression coefficients as $\beta ={\left(\beta_{0},\beta_{1}^{T}\right)}^{T}$ with $\beta_{1}=k\times {\left(0.5,0.3,0.3,0.25,0.25,0,0,0,0,0,0,0\right)}^{T}$, where $\beta_{0}$ and k are chosen to match the target outcome prevalence and *C*‐statistic, respectively. We consider two values for the (true) outcome prevalence, ϕ=0.1 or 0.5, and two values for the (true) *C*‐statistic, C=0.7 or 0.8. As the penalized methods were seen to demonstrate less variability in the CS than MLE when the predictors were highly correlated (Van Calster et al. 2020), we here focus on the less favorable scenario for penalized methods where correlations between predictors are weak. True predictors are weakly correlated with correlation 0.1, and noise predictors are weakly correlated with correlation 0.05, as in previous investigations (Riley et al. 2021).

For each scenario, that is, for each combination of outcome prevalence and *C*‐statistic values, we consider four possible sample sizes for the development datasets. The recommended sample size, N, was taken to be the sample size required according to the criterion of limiting overfitting, with a target expected shrinkage of 0.9 when MLE is used. We note that the sample size equation based on limiting model overfitting underestimated the sample size for the highest *C*‐statistic scenario, and so the sample size corresponding to an expected shrinkage of 0.9 was calculated using simulation. We also note that this sample size was larger than the size based on the other criteria for most of the scenarios we considered (Riley et al. 2019). We considered the recommended sample size and sample sizes close to it, that is: $\frac{N}{2},\frac{3}{4}N,N$ and $\frac{5}{4}N$. The R code for the main simulations is provided in Supplementary Material 2 and in the GitHub repository https://github.com/mpavlou/Improved‐tuning‐for‐penalised‐regression‐methods.

### 4.2 Simulation Results

Here we present results for the scenario with true *C*‐statistic = 0.7, true prevalence = 0.5, and 5 true and 7 noise predictors. We used $\beta_{0}=0$ and k=0.93 to obtain the vector of regression coefficients, and the recommended sample size was N=900. Detailed results for the other scenarios are presented in Supplementary Material 1.

#### 4.2.1 Tuning and Calibration Slope for Different Numbers of Cross‐Validation Folds

**FIGURE 1:**
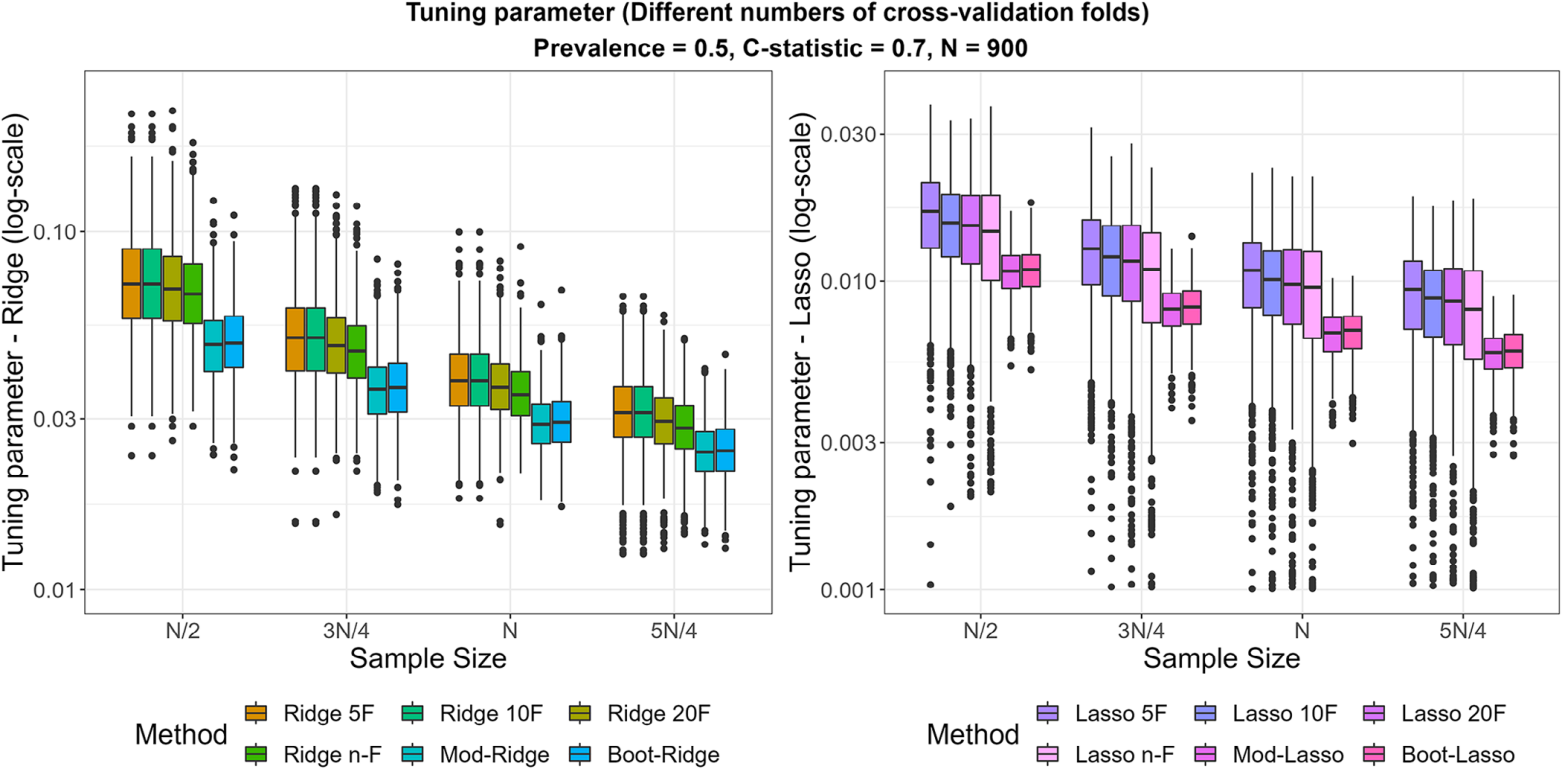
Estimated tuning parameters for the standard tuning (with different numbers of cross‐validation folds: Ridge 5‐fold (5F), 10F, 20F, *n*‐F, and similarly for Lasso) and modified or bootstrap tuning (Mod‐Ridge/Boot‐Ridge and Mod‐Lasso/Boot‐Lasso) over 1000 simulations. True *C*‐statistic = 0.7, true prevalence = 0.5.

Figure 1 shows that the median values of the tuning parameter selected using the modified or bootstrap tuning methods (Mod‐Ridge/Boot‐Ridge and Mod‐Lasso/Boot‐Lasso) are smaller than those from the corresponding standard tuning methods (Ridge and Lasso). In addition, there is less variability in the values of the selected tuning parameters for modified and bootstrap tuning.

**FIGURE 2:**
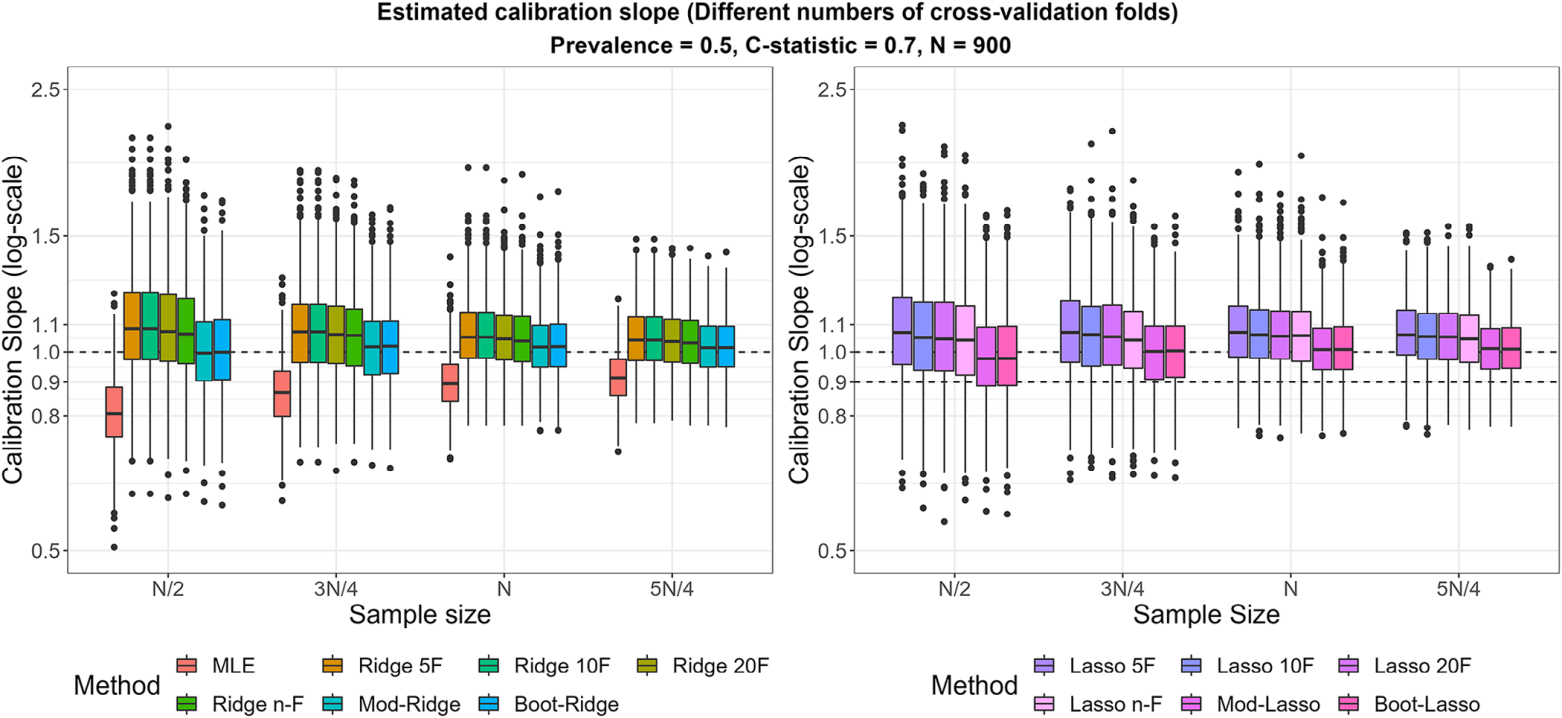
Calibration slope for the standard tuning (with different numbers of cross‐validation folds: Ridge 5‐fold (5F), 10F, 20F, *n*‐F, and similarly for Lasso) and modified or bootstrap tuning (Mod‐Ridge/Boot‐Ridge and Mod‐Lasso/Boot‐Lasso) over 1000 simulations. True *C*‐statistic = 0.7, true prevalence = 0.5. MLE, maximum likelihood estimation.

Increasing the number of cross‐validation folds for standard tuning resulted in a reduction in the magnitude and variability of the selected tuning parameters. However, this reduction was small compared to using the modified or bootstrap tuning, and its effect in terms of improving calibration was also small (Figures 1 and 2). This was also the case for *n*‐fold cross‐validation (where *n* is the size of the development sample), which, although on average selected a smaller λ than 5‐, 10‐, and 20‐fold cross‐validation, still led to a higher λ and worse calibration than the modified and bootstrap tuning, especially for Lasso. This could be probably attributed to the fact that although each of the training CV samples in *n*‐fold cross‐validation are of size *n*‐1, the test CV samples consist of only one data point, which may result in increased variability in the estimation of the loss‐function being minimized compared to having a larger cross‐validation sample. Overall, the modified and bootstrap tuning methods had almost identical performance.

#### 4.2.2 Predictive Performance on Average

**FIGURE 3:**
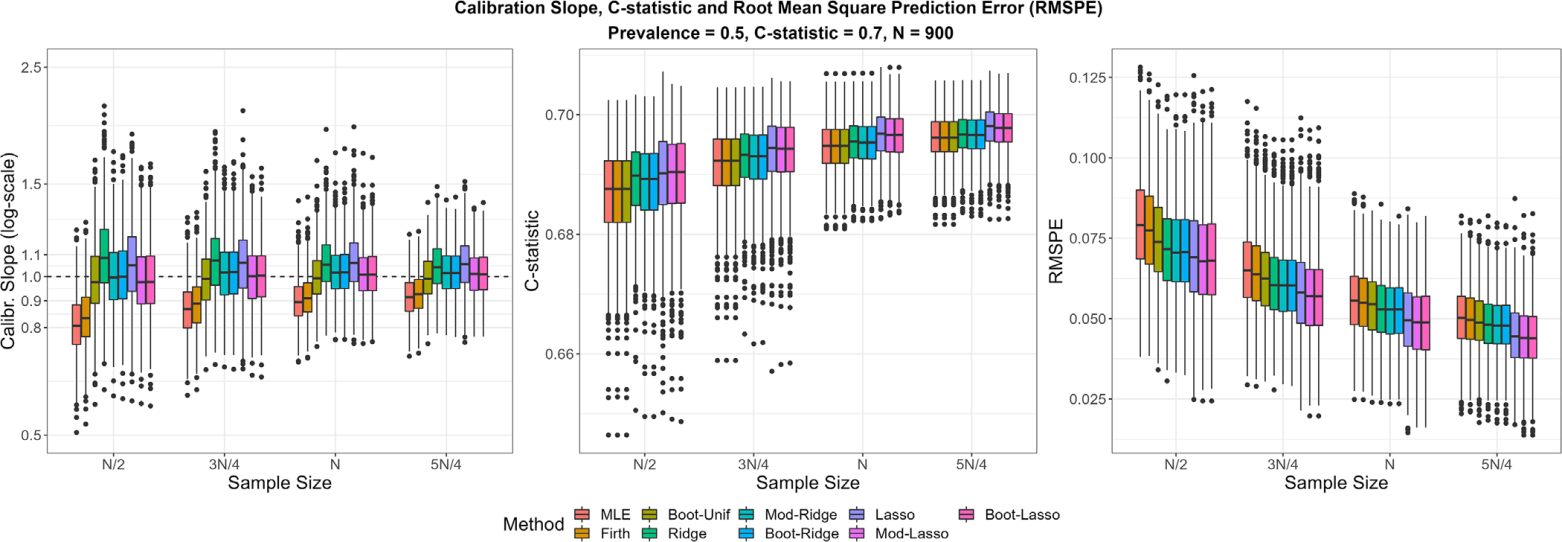
Calibration slope, *C*‐statistic, and root mean square prediction error (RPMSE) over 1000 simulations. True *C*‐statistic = 0.7, true prevalence = 0.5. MLE, maximum likelihood estimation.

Figure 3 summarizes the predictive performance of the methods in terms of calibration (CS), discrimination (*C*‐statistic), and overall predictive accuracy (RMSPE). As expected, the predictive performance of all methods improved with increasing sample size.

In terms of the CS, MLE produced overfitted models in the lowest sample size scenario, and the degree of overfitting reduced with increasing sample size. Firth's method had relatively low variability, but the shrinkage applied was on average very small, offering only a small improvement over MLE. Uniform shrinkage improved calibration on average compared to MLE, and its variability was slightly higher. Penalized methods, either with standard or modified tuning, improved calibration on average compared to MLE. However, as in previous studies, standard Ridge and Lasso tended to over‐shrink, particularly in the lowest sample size scenario, and to result in increased variability in the CS compared to MLE. Ridge and Lasso with modified and bootstrap tuning had CS closer to 1, on average, and the variability in CS was smaller than the standard Ridge and Lasso. These observations suggest that the modified and bootstrap tuning worked as expected. In terms of discrimination and predictive accuracy, Ridge and Lasso with either standard or modified/bootstrap tuning resulted in slightly better performance (also with slightly smaller variability) than MLE, Firth's method, and uniform shrinkage.

#### 4.2.3 Variability in Predictive Performance

These results suggest that modified and bootstrap tuning worked well, although the variability in the CS for Mod/Boot‐Ridge and Mod/Boot‐Lasso is still higher than that for MLE. To establish how Mod/Boot‐Ridge and Mod/Boot‐Lasso compare with MLE in terms of calibration for individual datasets, we consider performance measures that combine bias and variability in the CS.

**FIGURE 4:**
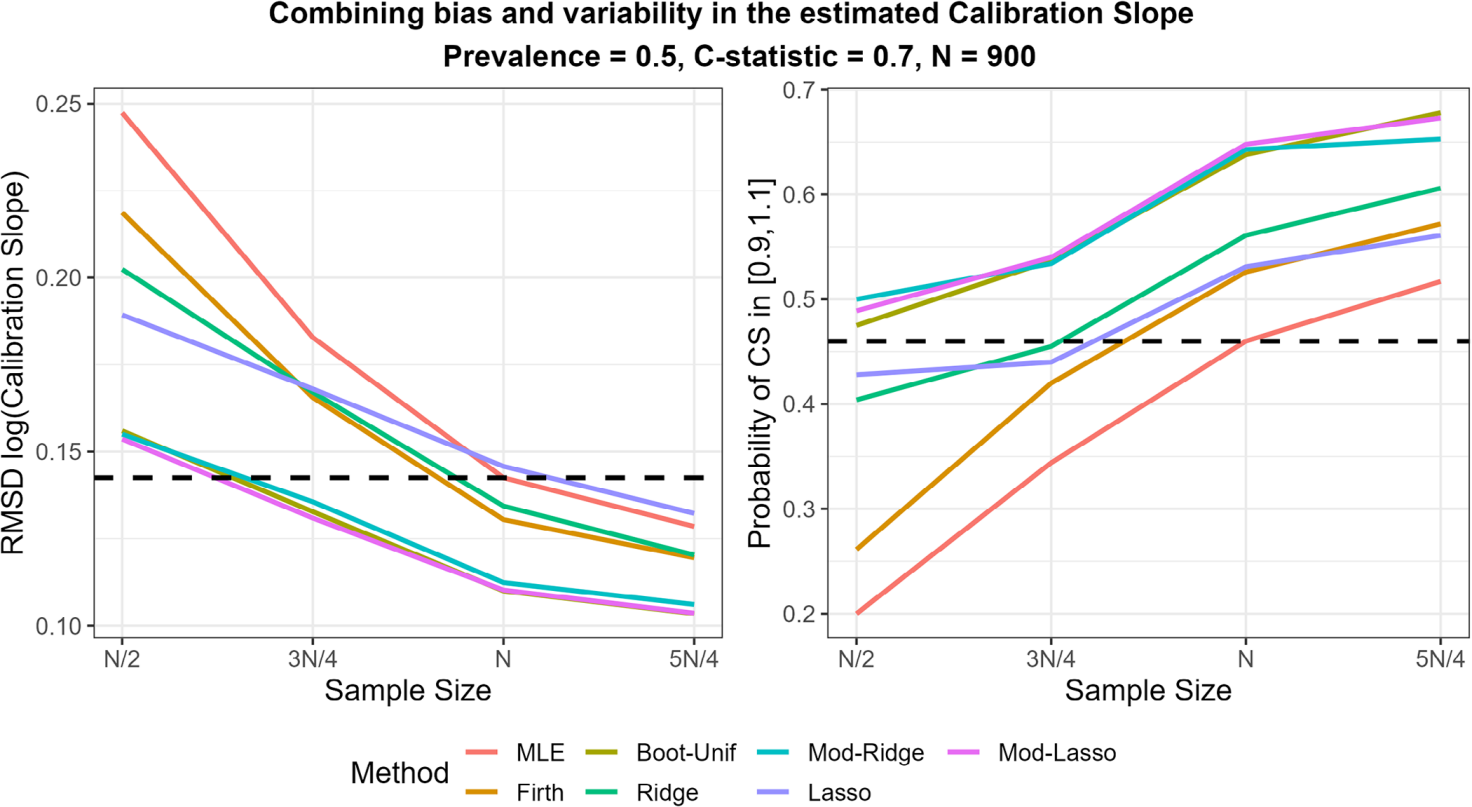
Root mean squared distance for the log‐calibration slope and probability of obtaining a well‐calibrated model (calibration slope between 0.9 and 1.1) over 1000 simulations. The dashed horizontal lines correspond to the performance of MLE at the recommended sample size. The results for Boot‐Ridge (not shown) were effectively identical to Mod‐Ridge. True *C*‐statistic = 0.7, true prevalence = 0.5. MLE, maximum likelihood estimation.

As the results for modified and bootstrap tuning were very similar, for the clarity of presentation in the main paper, we present results just for the first (results for bootstrap tuning are in Figure S1). Figure 4 shows the RMSD(log‐CS) and the probability of obtaining a well‐calibrated model. It is clear that modified tuning resulted in substantially lower RMSD(log‐CS) and a higher probability of providing a well‐calibrated model than MLE for a given sample size. For instance, at the recommended sample size (N=900), the RMSD(log‐CS) for MLE was just under 0.15. The same RMSD(log‐CS) was achieved at N≈600 when either Mod‐Ridge, Mod‐Lasso, or Boot‐Unif were used. Moreover, the probability of obtaining a well‐calibrated model at the recommended sample size, N=900, was 46% for MLE and around 65% for Mod‐Ridge, Mod‐Lasso, and Boot‐Unif. When the sample size was 680, 25% smaller than the recommended, Mod‐Ridge, Mod‐Lasso, and Boot‐Unif still led to a well‐calibrated model a considerable 53% of the time, whereas MLE only 35%. These results suggest that Mod‐Ridge, Mod‐Lasso, and Boot‐Unif have the potential to increase the chance of obtaining a well‐calibrated model compared to MLE.

Finally, when the sample size was 680, Boot‐Unif, Mod‐Ridge, and Mod‐Lasso improved calibration compared to MLE (i.e., led to a value of CS closer to 1 in individual datasets) 73%, 69%, and 71% of the time, respectively (Figure S2). Moreover, Mod‐Ridge and Mod‐Lasso were almost guaranteed to improve discrimination (99% and 94% of the time, respectively) and had a high probability of improving predictive accuracy (80% and 91%, respectively) compared to MLE in individual datasets (Figures S3 and S4).

The results were very similar for scenarios based on other prevalence and *C*‐statistic values (Figures S5–S7). It is worth noting that the sample size for which penalized methods may lead to a reasonably good model depends on the true model strength (*C*‐statistic) and outcome prevalence and is closely linked to the recommended sample size (Riley et al. 2019). For example, when C=0.7 and ϕ=0.5, the recommended sample size was 900. Considering both bias and variability in the estimated CS, Boot‐Ridge and Boot‐Lasso provided results comparable to MLE in terms of model calibration for sample size down to around 600 (Figure 4). For C=0.8 and ϕ=0.1 (recommended sample size = 1120), they provided results comparable to MLE with sample size down to 700 (Figure S7).

#### 4.2.4 Summary and Practical Implications

Several studies used simulation to explore the performance of standard Ridge and Lasso for risk prediction. They suggested that these methods should not be used at the recommended sample sizes or lower, because even though they improved calibration performance on average, they performed worse than MLE when variability was taken into account. We have considered simulation scenarios very similar to those studies, and we have found that Ridge and Lasso, with modified or bootstrap tuning, resulted in improved calibration compared to MLE at sample sizes close to those recommended. Specifically, there was a higher probability of providing a well‐calibrated model with a smaller RMSD(log‐CS) compared to Ridge and Lasso with standard tuning and MLE. They also offered small gains with respect to the *C*‐statistic and RPMSE compared to MLE. Hence, there seems to be a benefit of using Ridge and Lasso with modified or bootstrap tuning methods in practice, at least in settings similar to those considered in our evaluation.

In practice, the sample size for a prediction study should *always* be chosen to be at least as large as those recommended by recent guidelines (Riley et al. 2019). The recommended sample size is typically calculated *before data collection and model fitting*. Its calculation is informed by the number of candidate predictor variables and the anticipated values of the *C*‐statistic and outcome prevalence. When the sample size of the development data matches the recommended sample size or is slightly larger, Ridge and Lasso with modified or bootstrap tuning can perform better than MLE and Ridge and Lasso with standard tuning. In the unfortunate situation where the development sample size is too small, for example, because the anticipated values for the *C*‐statistic and/or outcome prevalence were set too high, Ridge and Lasso with either modified or bootstrap tuning may help in alleviating model overfitting and providing more stable models than MLE.

## 5 Real Data Illustration

In this section, we consider real cardiac data from patients undergoing heart valve surgery (Ambler et al. 2005) to assess the performance of the modified and bootstrap tuning methods. In this illustration, we use data on 16,679 patients in Great Britain and Ireland who had heart valve surgery between 1995 and 2003. The outcome of interest is in‐hospital death (binary outcome) following heart valve surgery (prevalence 7%). The aim is to develop a risk model to predict the risk of in‐hospital death. We considered a mixture of 11 binary and continuous variables, which are described in Table S1.

To evaluate the performance of the methods, we sampled patients without replacement from the original dataset to form a training dataset with the desired sample size; the remaining data form a validation dataset. We fit logistic regression models in the training dataset using MLE, standard Ridge, and Ridge with the modified and bootstrap tuning methods. We then validated each estimated model on the validation dataset. This process was repeated 200 times for each sample size. We then focused on assessing the bias and variance in the CS using the metrics used in the simulation study. We considered 3 sample sizes, the middle of which (2200 patients) corresponds to the CS being 0.9 on average for MLE (this would be the recommended size).

**FIGURE 5:**
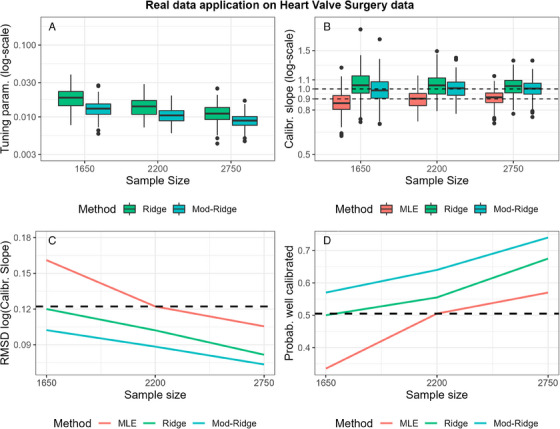
Data from heart valve surgery: illustration of the application of ridge regression with the modified tuning method when developing a risk model (A: tuning parameter; B: calibration slope; C: RMSD(log‐CS); D: probability of well‐calibrated model). The dashed horizontal lines in (C) and (D) correspond to the performance of MLE at the recommended sample size. MLE, maximum likelihood estimation; RMSD, root mean square distance.

The results from the real data application support the conclusions of the simulation study. As it can be seen in Figure 5, the modified tuning method (results for bootstrap tuning were effectively identical) substantially reduces the variability and size of the chosen tuning parameter compared to the standard tuning method (Figure 5A). This is beneficial in terms of the CS, which is closer to one on average (and with smaller variance) for the modified than the standard tuning method (Figure 5B). When considering both the bias and variance of the CS (Figure 5C,D), Mod‐Ridge has a lower RMSD(log‐CS) and a higher probability of providing a well‐calibrated model (CS between 0.9 and 1.1) than MLE for the sample sizes considered. Similar to the simulation study, both Ridge and Mod‐Ridge modestly improved discrimination and predictive accuracy (Brier Score), with the classical ridge performing marginally better (Figure S8). In Supplementary Material 2, we include code for reproducible analysis based on synthetic data that resemble the real data. The results are shown in Figures S9 and S10, and the conclusions drawn are similar to those for the real data and the main simulation study.

## 6 Discussion

Previous studies investigated whether Ridge and Lasso regression could reduce model overfitting. Although they improved calibration on average, they were found to over‐shrink and lead to high variability in the CS (Riley et al. 2021; Šinkovec et al. 2021; Van Calster et al. 2020). Consequently, they were found to offer little or no advantage over MLE when considering both bias and variability. This applied to both datasets of the recommended sample size to limit model overfitting and smaller.

A possible reason for the high variability in the CS is the uncertainty in the selection of a suitable shrinkage (tuning) parameter. The latter is typically selected using cross‐validation to maximize the out‐of‐sample predictive performance of the model. As the datasets involved in cross‐validation are typically smaller than the development dataset, the tuning parameter tends to be overestimated, resulting in excessive shrinkage, especially for small datasets.

We have proposed a simple method to improve the selection of the tuning parameter, by ensuring that the cross‐validation training datasets are of the same size as the original development dataset. We achieve this by selecting the tuning parameter on a pseudo‐development dataset that is larger than the original, such that the resulting cross‐validation training datasets are of the same size as the development dataset. The pseudo‐development dataset is obtained by sampling with replacement from the original dataset. This approach is closely related to the standard bootstrap tuning selection of the tuning parameter, which we also considered.

Our simulation studies have shown that the modified and bootstrap tuning methods can reduce not only the magnitude of the chosen tuning parameter but also the uncertainty around the chosen value. As a result, modified and bootstrap tuning resulted in the CS being closer to the target value of 1 on average, and with smaller variability than that produced by the standard tuning method. In comparison to MLE, Ridge and Lasso with modified and bootstrap tuning also had a lower RMSD(log‐CS) and a higher probability of producing a well‐calibrated model at the recommended sample sizes to limit overfitting and slightly smaller or higher. In our simulation studies as well as the real data application we considered, penalized regression with the modified and bootstrap tuning methods provided comparable calibration results to MLE with sample sizes at least 25% smaller. Despite this, we suggest as a conservative approach that penalized regression with either modified or bootstrap tuning methods always be used in conjunction with the recommended sample size requirements according to the criterion of limiting overfitting and other criteria (Riley et al. 2020).

One limitation of Ridge and Lasso, even with the modified and bootstrap tuning, is that although they have led to a higher probability of obtaining a well‐calibrated model compared to MLE in the scenarios considered, they did not improve calibration compared to MLE in every single individual dataset. This is because although on average the CS for MLE may be close to, for example, 0.9 for an adequately chosen size, across samples it will vary around that value. For values of the CS for MLE‐fitted models in individual datasets that are close to the expected CS or lower, applying some shrinkage via penalized methods with modified tuning tends to improve calibration compared to MLE (CS closer to 1). However, for some individual datasets, the CS for MLE‐fitted models will be close to 1 or may even exceed this value (see, e.g., Figure 3). In those cases, applying shrinkage will tend to push the CS further away from 1, though this may not necessarily lead to a poorly calibrated model. On the other hand, in terms of discrimination and predictive accuracy, for a given sample size, Ridge and Lasso with modified or bootstrap tuning were almost guaranteed to provide a model with a higher *C*‐statistic and lower RMSPE than MLE.

A limitation of Ridge and Lasso is that they tend to apply little or no shrinkage when separation occurs and, hence, fail to solve overfitting problems on those occasions (Šinkovec et al. 2021). As our modified tuning method tends to apply less shrinkage than standard tuning, it should not be the method of choice either for separated datasets. Instead, when separation is detected in a dataset, we recommend the use of methods that are specifically designed to handle separation, such as Firth's method.

To conclude, the modified tuning method we proposed is easy to apply and should be conservative (i.e., applies less shrinkage than the standard tuning methods). It was seen to have almost identical performance to bootstrap tuning in simulations. Penalized methods with either modified and bootstrap tuning have the potential to produce better models at the recommended sample sizes than MLE and penalized methods with standard tuning. Moreover, they may produce comparable models to MLE in terms of predictive performance, with smaller sample sizes.

## Conflicts of Interest

The authors declare no conflicts of interest.

## Open Research Badges

This article has earned an Open Data badge for making publicly available the digitally‐shareable data necessary toreproduce the reported results. The data is available in the Supporting Information section.

This article has earned an open data badge “Reproducible Research” for making publicly available the code necessaryto reproduce the reported results. The results reported in this article were reproduced partially due to confidentiality issues.

## Supporting information

Supporting Information

Supporting Information

## Data Availability

The data that support the findings of this study were used under license for the current study and are therefore not publicly available. The data are secondary data (obtained from the database of the Society of Cardiothoracic Surgeons of Great Britain and Ireland (SCTS) for April 1995 to March 2003). R code that generates the data used for the simulation studies and synthetic data with features that resemble the features of the real data are provided in Supplementary Material 2.
