# Supplementary material for: Penalized Regression Methods With Modified Cross‐Validation and Bootstrap Tuning Produce Better Prediction Models
Source: Biom J. 2024 Jun 24;66(5):e202300245. doi: 10.1002/bimj.202300245 (PMC12859537; doi:10.1002/bimj.202300245)
Supplement: Supplementary file 1 — Supporting Information [file BIMJ-66-e202300245-s001.docx]

Supplementary Material 1

# Supplementary Figures

#### Figure S1

Comparison of ‘Bootstrap tuning’ to ‘Modified tuning’ in terms of measures combining bias and variability in the calibration slope.


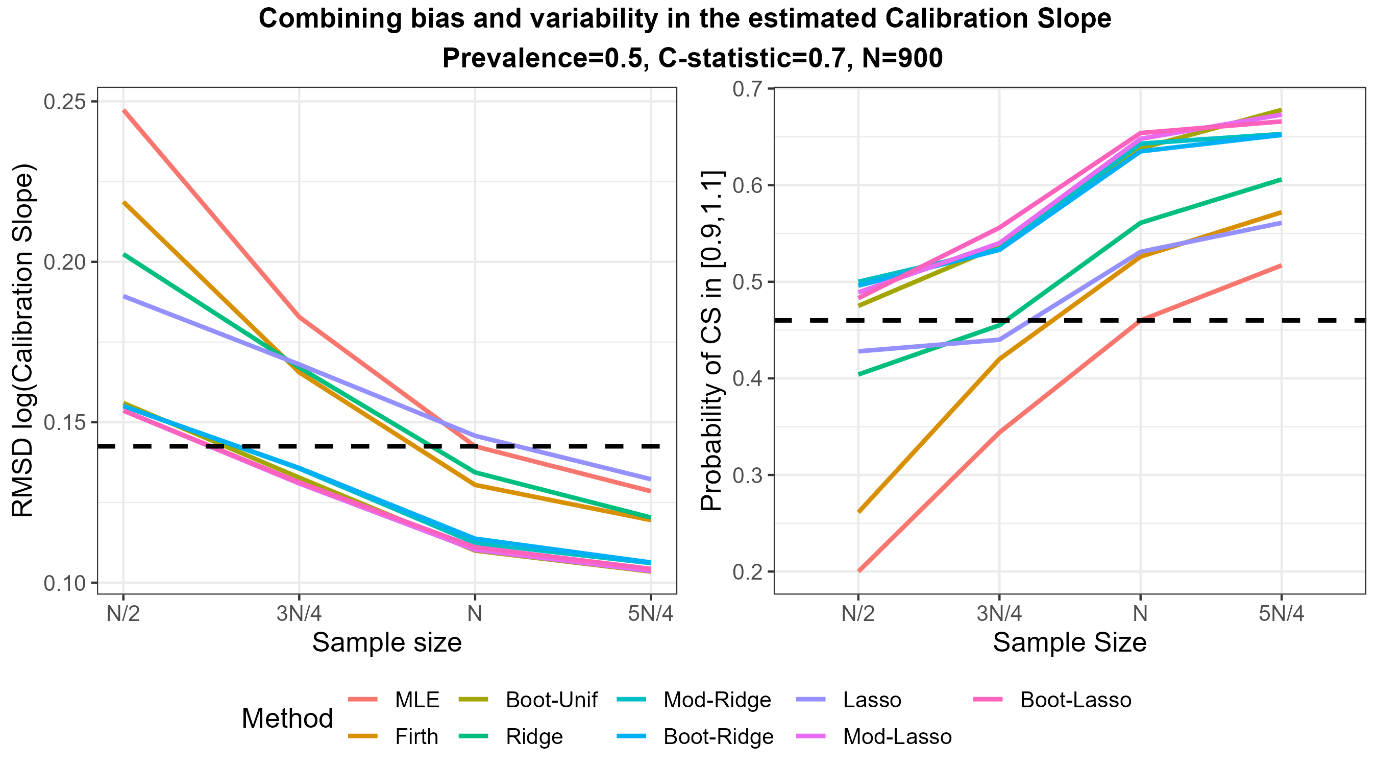


#### Figure S2

Calibration slope for a given method (y-axis) against the calibration slope for MLE. The number on the top of each graph is the proportion of times (over 1000 simulations) that the method on the y-axis is better than MLE.


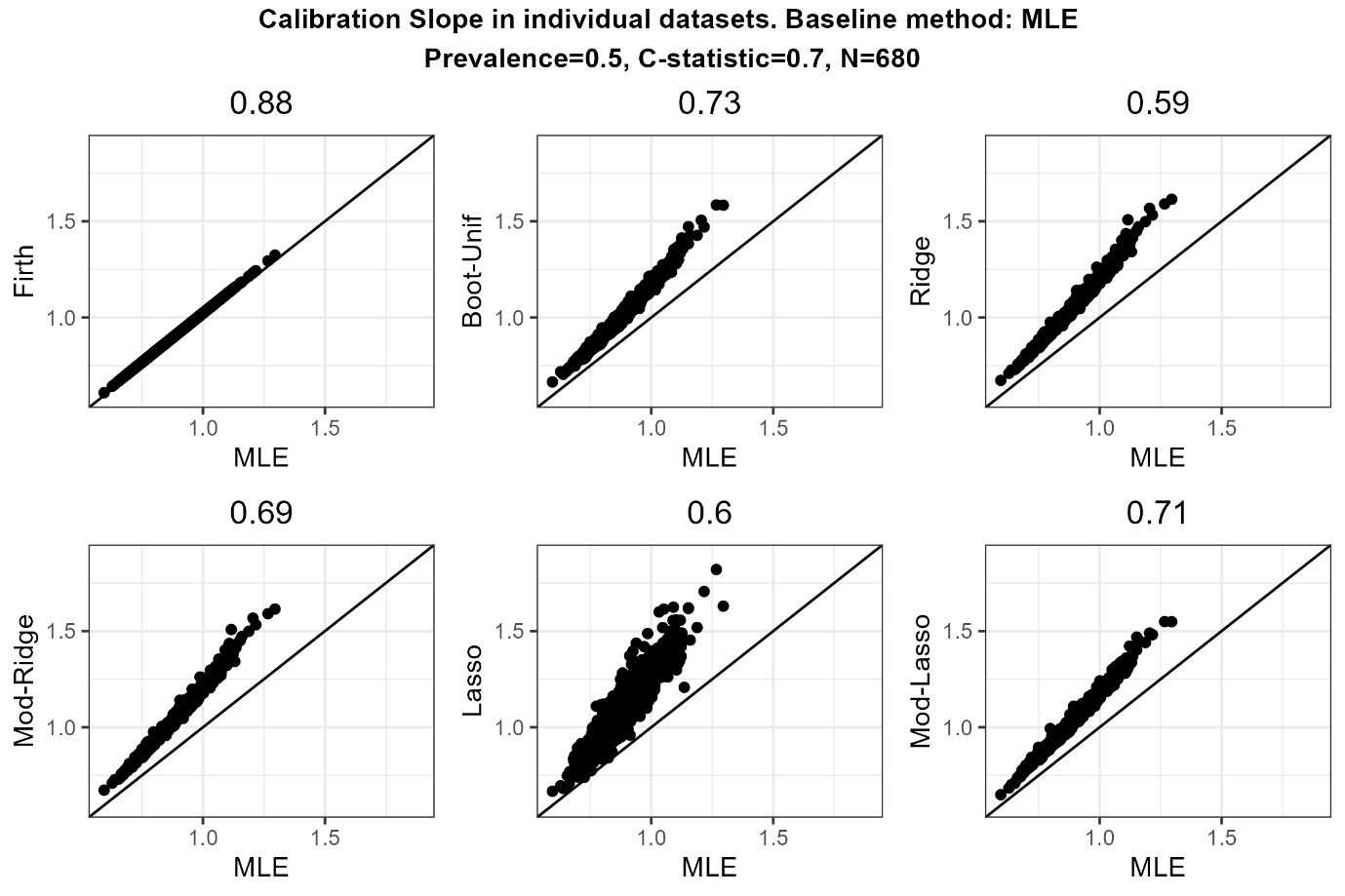


#### Figure S3

Estimated C-statistic for a given method (y-axis) against the C-statistic for MLE (same as Boot-Unif). The number of top of each graph is the proportion of times (over 1000 simulations) that the method on the y-axis is better than MLE.


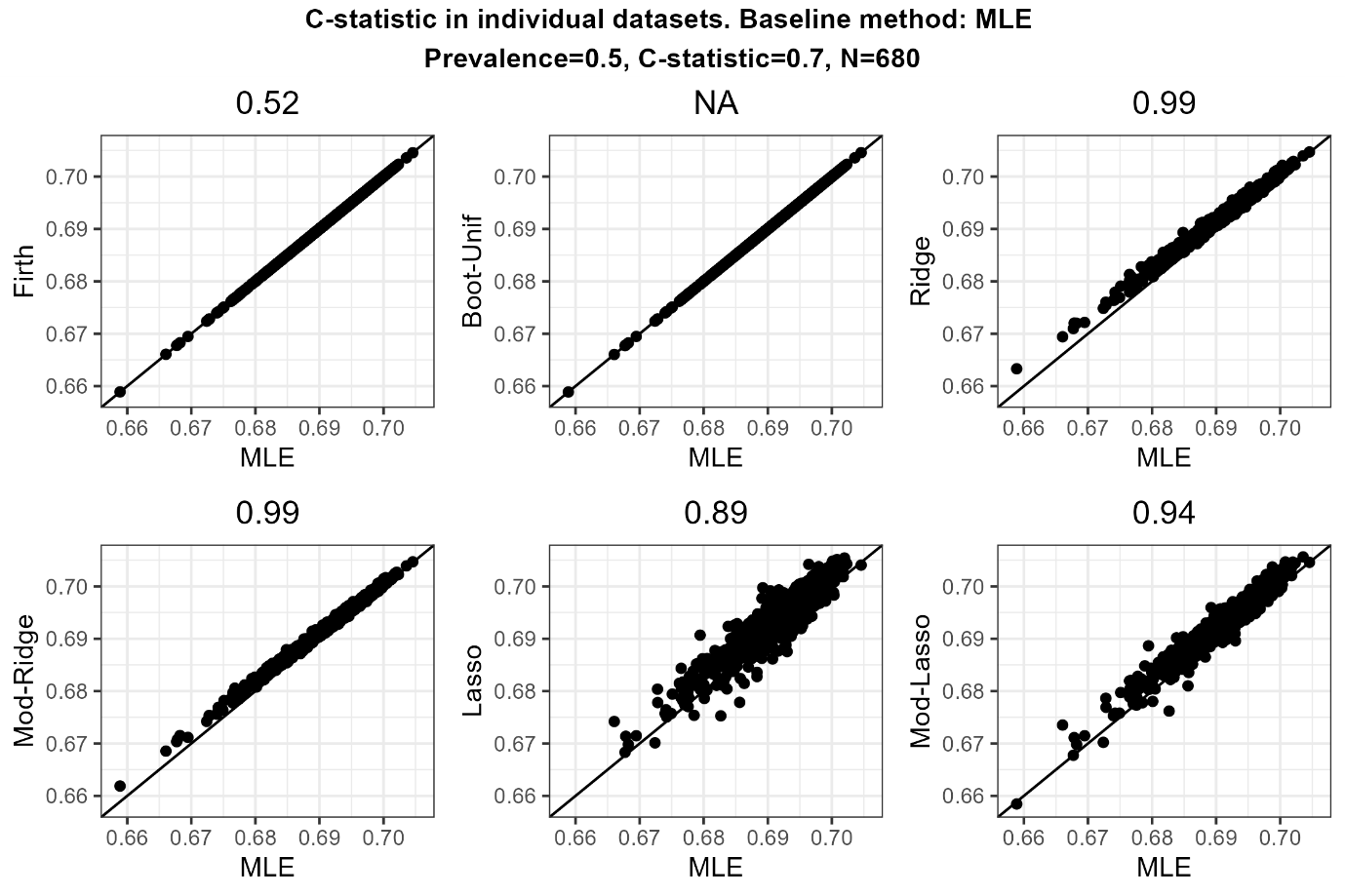


#### Figure S4

Estimated Root Mean Square Prediction Error (RPMSE) for a given method (y-axis) against the RPMSE for MLE. The number of top of each graph is the proportion of times (over 1000 simulations) that the method on the y-axis is better than MLE.


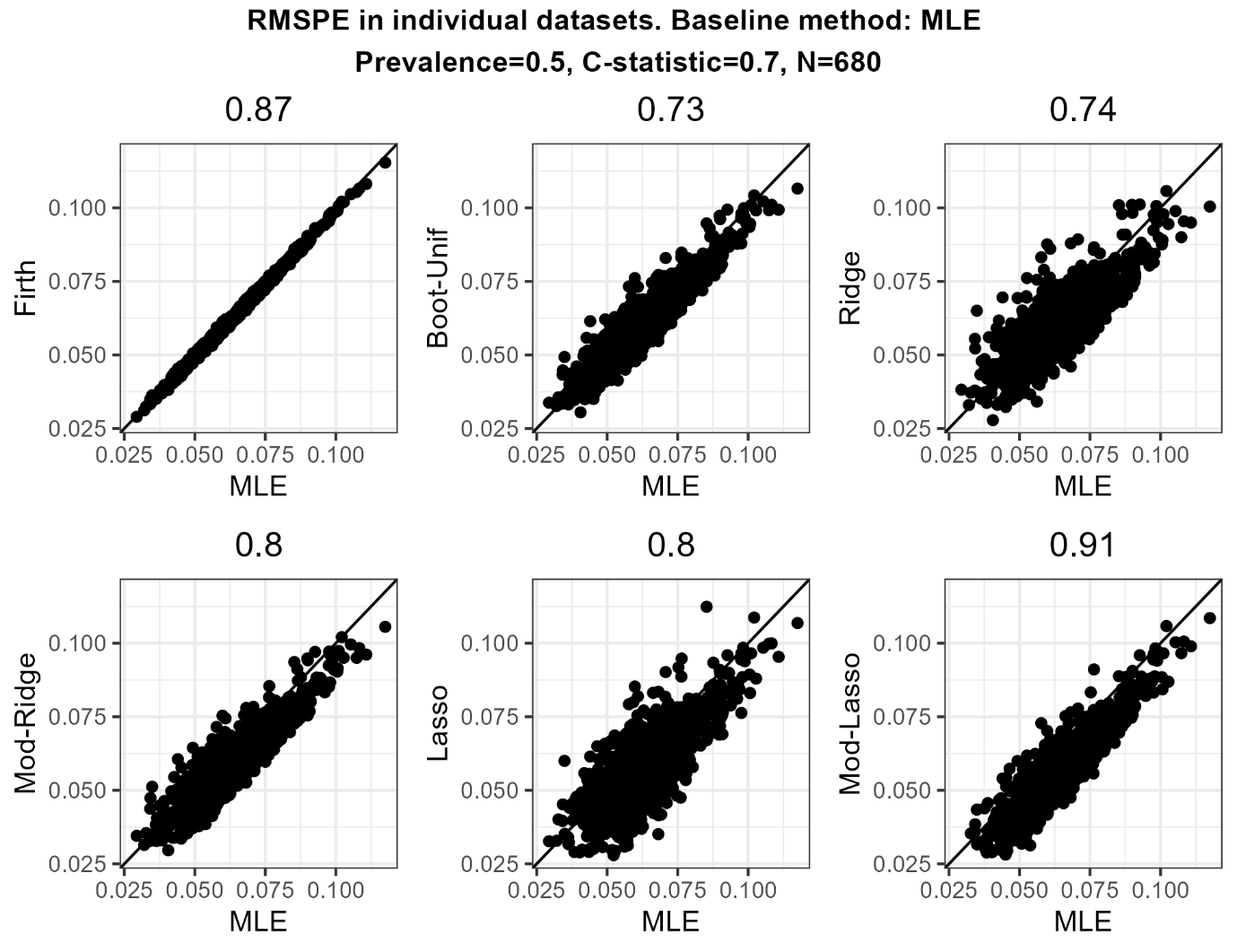


#### Figure S5

Calibration Slope, Root Mean Square Prediction Error, Root Mean Squared Distance for the log-Calibration Slope and Probability of obtaining a well-calibrated model (calibration slope between 0.9 and 1.1) over 1000 simulations. True C-stat=0.8, true prevalence=0.5.


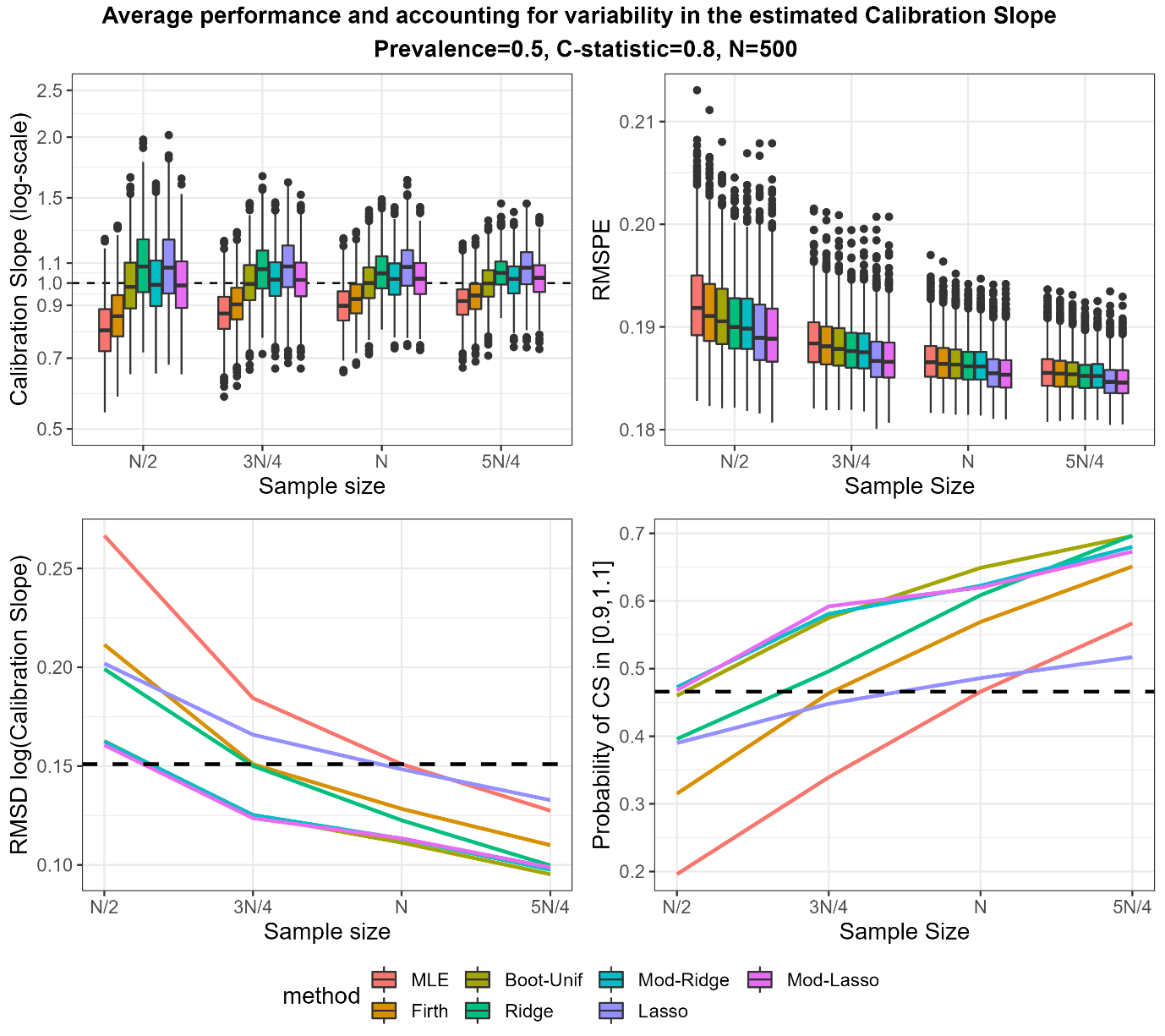


#### *Figure S6*

Calibration Slope, Root Mean Square Prediction Error, Root Mean Squared Distance for the log-Calibration Slope and Probability of obtaining a well-calibrated model (calibration slope between 0.9 and 1.1) over 1000 simulations. True C-stat=0.7, true prevalence=0.1.


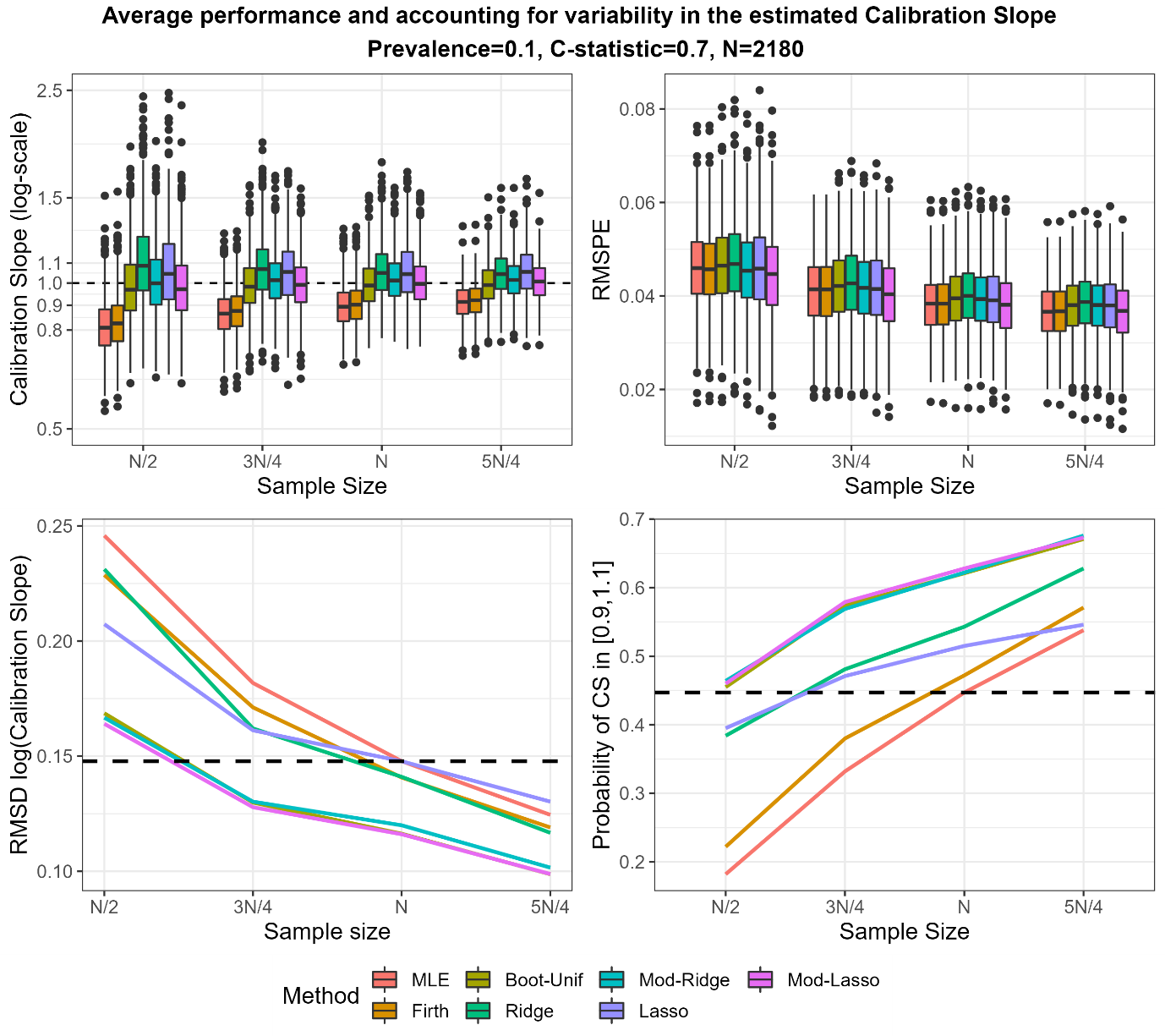


#### Figure S7

Calibration Slope, Root Mean Square Prediction Error, Root Mean Squared Distance for the log-Calibration Slope and Probability of obtaining a well-calibrated model (calibration slope between 0.9 and 1.1) over 1000 simulations. True C-stat=0.8, true prevalence=0.1.

####
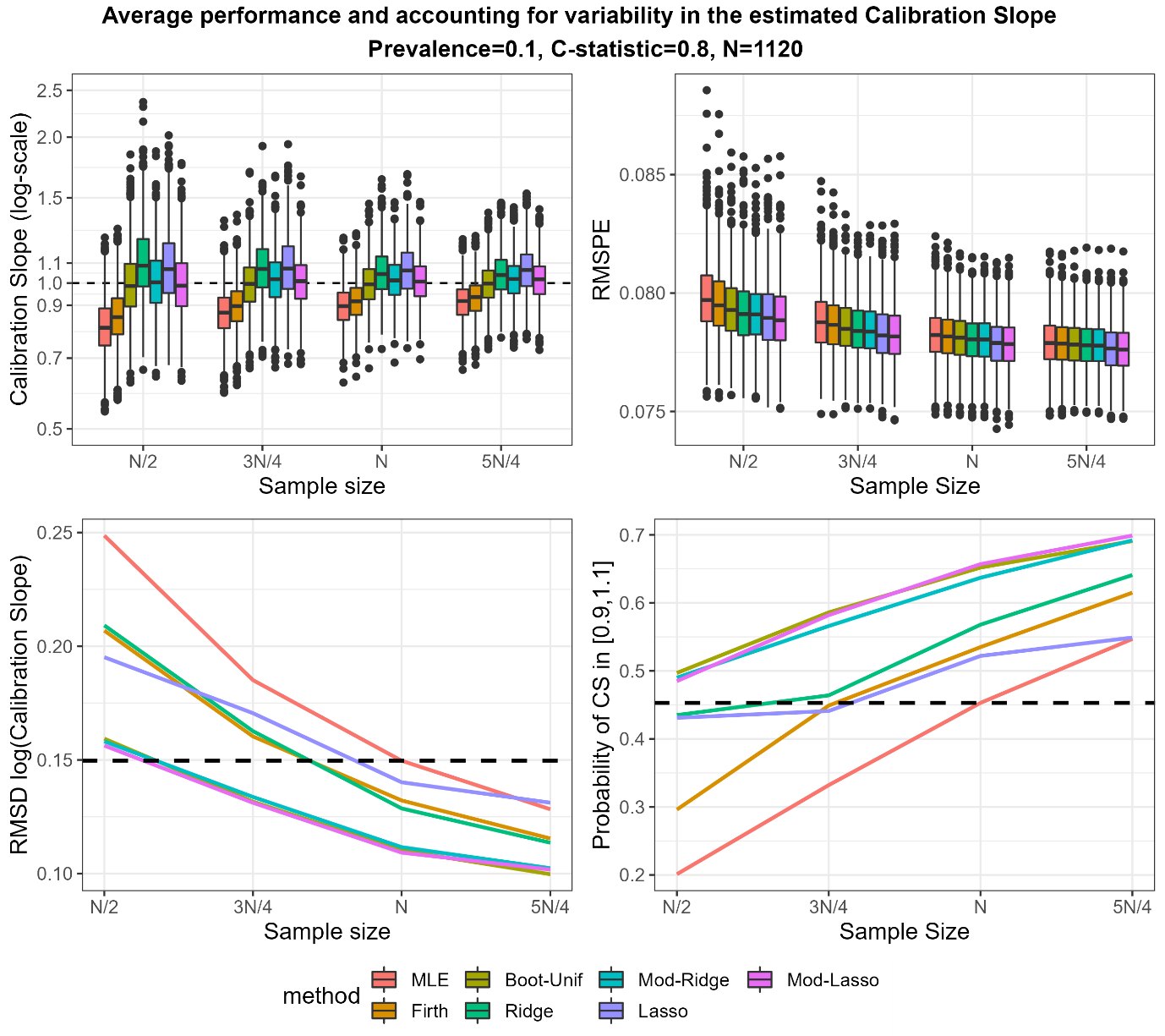
Figure S8

Real data from heart valve surgery: illustration of the application of ridge regression with the modified tuning when developing a risk model (C-statistic and Brier score).


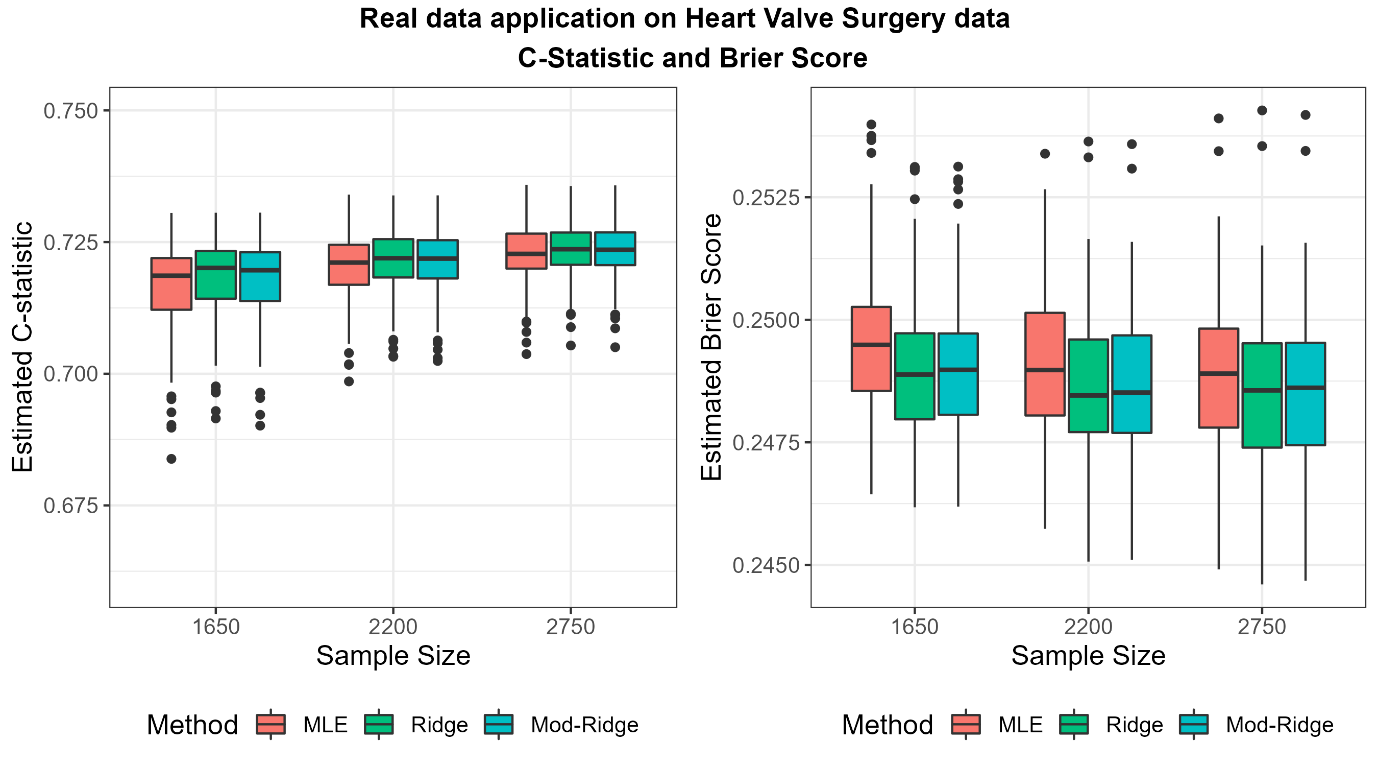


#### Figure S9

Synthetic data from heart valve surgery: illustration of the application of ridge regression with the modified tuning when developing a risk model (A: tuning parameter; B: calibration slope; C: RMSD; D) Probability of well calibrated model).


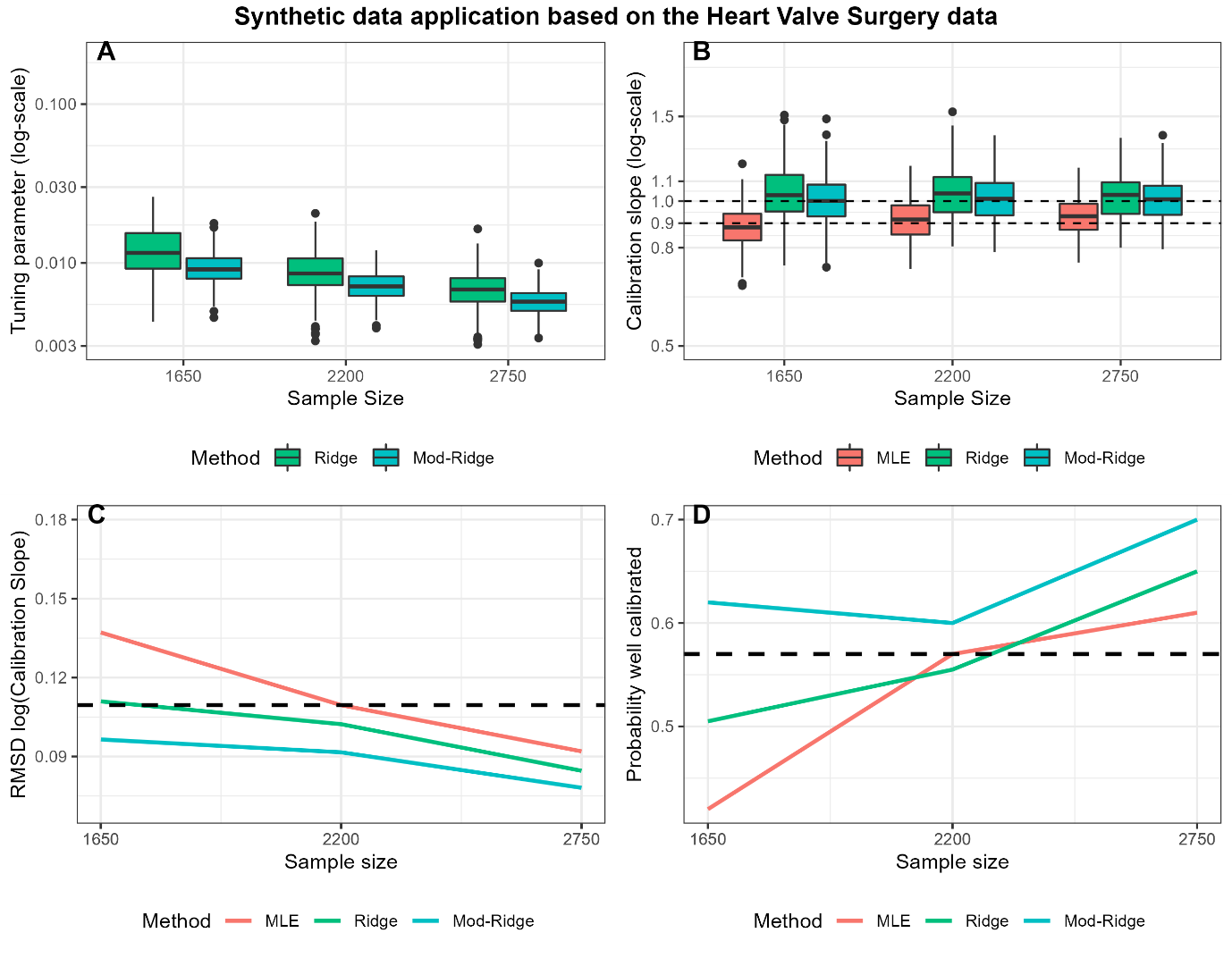


#### Figure S10

Synthetic data from heart valve surgery: illustration of the application of ridge regression with the modified tuning when developing a risk model (C-statistic and Brier score).


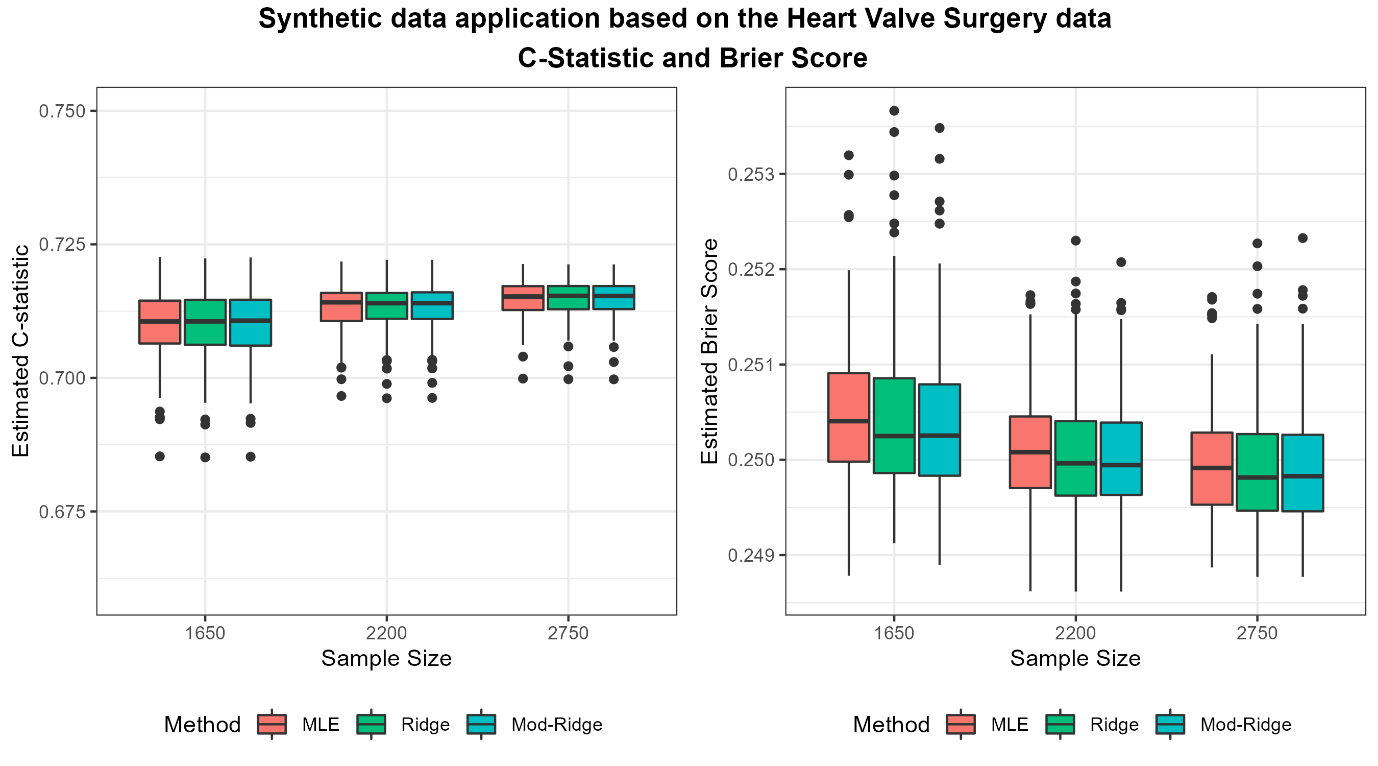


|  | **Mean/N** | **SD/Proportion** |
| --- | --- | --- |
|  |  |  |
| Age *(‘age’)* | 64.4 | 12.2 |
| BMI *(‘bmi’)* | 25.3 | 4.85 |
|  | | |
| Female Sex -Yes (*‘sex’)* | 6955 | 0.42 |
| Respiratory Disease - Yes (‘zresp’) | 861 | 0.05 |
| Renal Failure - Yes *(‘zrenal’)* | 1285 | 0.08 |
| Preoperative arrhythmias - Yes *(‘zarryth’)* | 5785 | 0.35 |
| Hypertension - Yes *(‘zhyper’)* | 5166 | 0.31 |
| Ejection function >50% - Yes *(‘zejec’)* | 1183 | 0.07 |
| Diabetes - Yes *(‘zdiab’)* | 1295 | 0.08 |
| Operative Priority - Urgent/Emergency *(‘zprior’)* | 4860 | 0.29 |
| Operation Sequence - Second or More *(‘seq’)* | 2356 | 0.14 |
|  |  |  |
| **In-Hospital death - Yes (‘*Status’*)** | 1163 | 0.07 |
|  |  |  |
|  |  |  |

# Real Data Application

We considered a mixture of eleven binary and continuous variables for inclusion in the model: operative priority (elective vs urgent or emergency), age in years (continuous), renal failure (no vs yes) , ejection fraction (poor (<30%) or fair ($\leq$50%) vs good (>50%), operation sequence (first vs second or more), respiratory disease (no vs yes), body mass index in kg/m^2^ (continuous), preoperative arrhythmias (no vs yes), diabetes (no vs yes), sex (male vs female), and hypertension (no vs yes). For binary predictors, the most prevalent category was taken to be the baseline.

#### Table S1

Descriptive Statistics for the variables in the real data application (Heart Valve Surgery). Based on 16679 patients. The outcome variable is in-hospital death.
