# Supplementary material for: Penalized Regression Methods With Modified Cross‐Validation and Bootstrap Tuning Produce Better Prediction Models
Source: Biom J. 2024 Jun 24;66(5):e202300245. doi: 10.1002/bimj.202300245 (PMC12859537; doi:10.1002/bimj.202300245)
Supplement: Supplementary file 2 — Supporting Information [file BIMJ-66-e202300245-s002.zip › Supplementary_Material_2/figures_tables/figure_2.pdf]

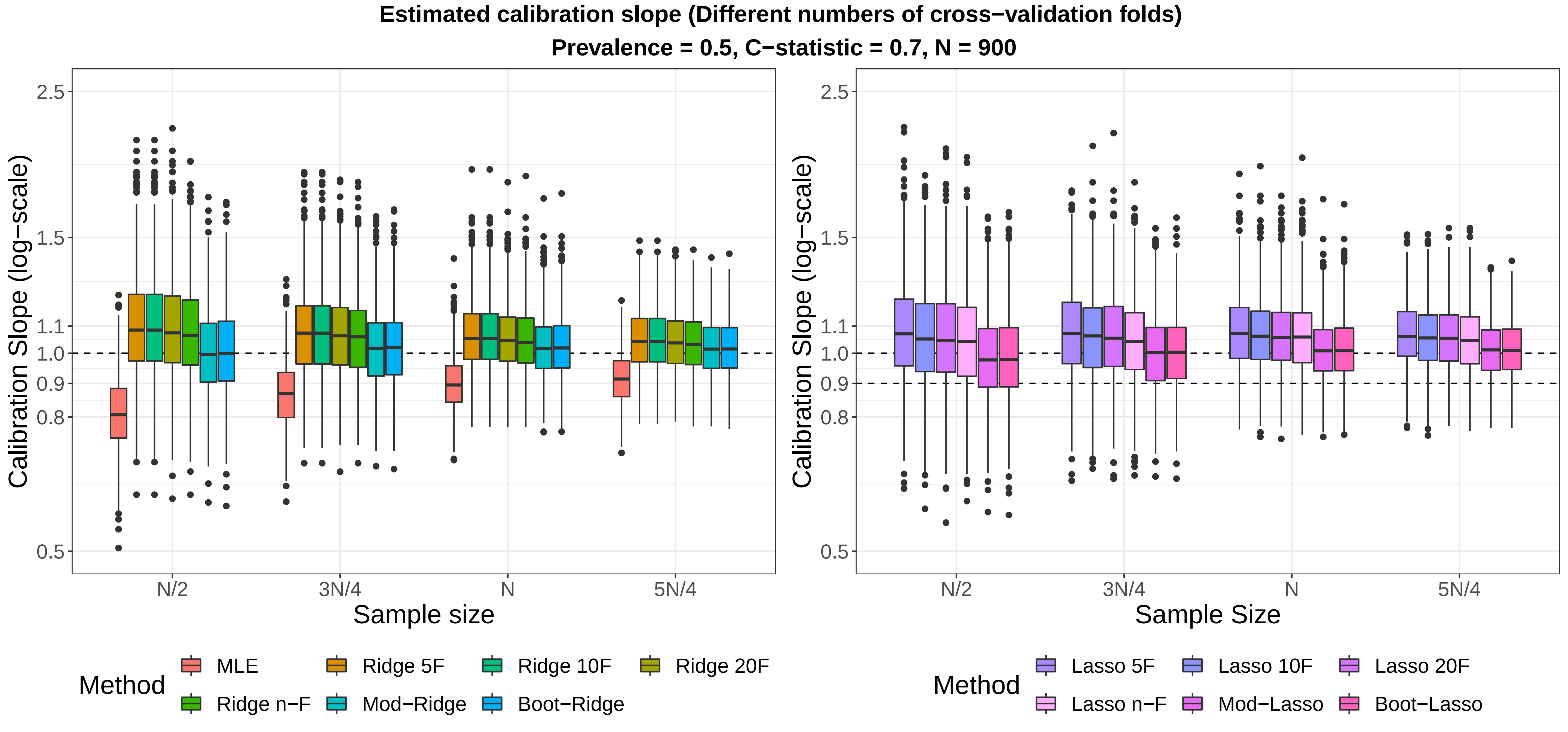
Estimated calibration slope (Different numbers of cross−validation folds)
Prevalence = 0.5, C−statistic = 0.7, N = 900
Calibration Slope (log−scale)
2.5
1.5
1.1
1.0
0.9
0.8
0.5
N/2
3N/4
N
5N/4
Sample size
Sample Size
Method
MLE
Ridge 5F
Ridge 10F
Ridge 20F
Ridge n−F
Mod−Ridge
Boot−Ridge
Lasso 5F
Lasso 10F
Lasso 20F
Lasso n−F
Mod−Lasso
Boot−Lasso
