# Supplementary material for: Penalized Regression Methods With Modified Cross‐Validation and Bootstrap Tuning Produce Better Prediction Models
Source: Biom J. 2024 Jun 24;66(5):e202300245. doi: 10.1002/bimj.202300245 (PMC12859537; doi:10.1002/bimj.202300245)
Supplement: Supplementary file 2 — Supporting Information [file BIMJ-66-e202300245-s002.zip › Supplementary_Material_2/figures_tables/figure_3.pdf]

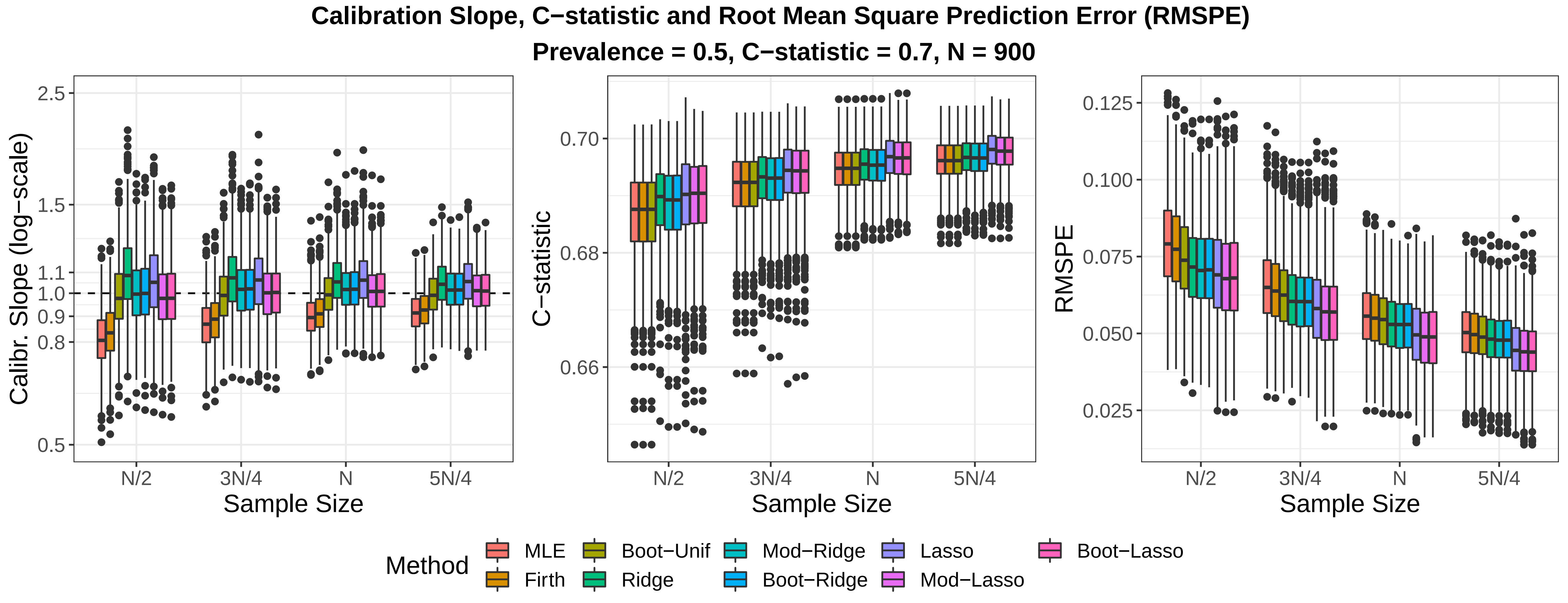

Calibration Slope, C−statistic and Root Mean Square Prediction Error (RMSPE)
Prevalence = 0.5, C−statistic = 0.7, N = 900
Calibr. Slope (log−scale)
2.5
1.5
1.1
1.0
0.9
0.8
0.5
C−statistic
0.70
0.68
0.66
RMSPE
0.125
0.100
0.075
0.050
0.025
N/2
3N/4
N
5N/4
Sample Size
Method
MLE
Firth
Boot−Unif
Ridge
Mod−Ridge
Boot−Ridge
Lasso
Mod−Lasso
Boot−Lasso
