# Supplementary material for: Penalized Regression Methods With Modified Cross‐Validation and Bootstrap Tuning Produce Better Prediction Models
Source: Biom J. 2024 Jun 24;66(5):e202300245. doi: 10.1002/bimj.202300245 (PMC12859537; doi:10.1002/bimj.202300245)
Supplement: Supplementary file 2 — Supporting Information [file BIMJ-66-e202300245-s002.zip › Supplementary_Material_2/figures_tables/figure_S10.pdf]

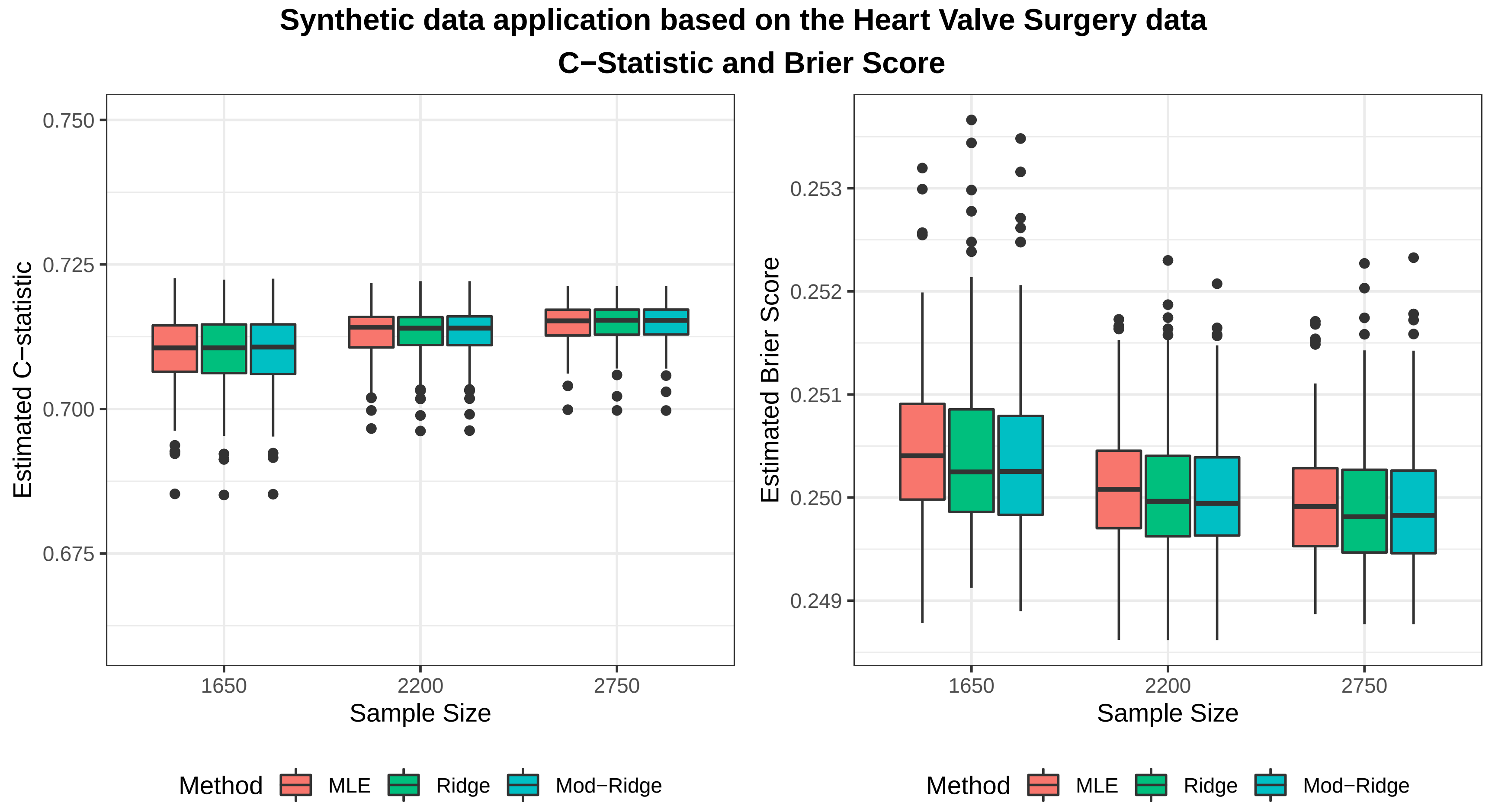

Synthetic data application based on the Heart Valve Surgery data
C−Statistic and Brier Score
Estimated C−statistic
0.750
0.725
0.700
0.675
1650
2200
2750
Sample Size
Method
MLE
Ridge
Mod−Ridge
Estimated Brier Score
0.253
0.252
0.251
0.250
0.249
1650
2200
2750
Sample Size
Method
MLE
Ridge
Mod−Ridge
