# Supplementary material for: Penalized Regression Methods With Modified Cross‐Validation and Bootstrap Tuning Produce Better Prediction Models
Source: Biom J. 2024 Jun 24;66(5):e202300245. doi: 10.1002/bimj.202300245 (PMC12859537; doi:10.1002/bimj.202300245)
Supplement: Supplementary file 2 — Supporting Information [file BIMJ-66-e202300245-s002.zip › Supplementary_Material_2/figures_tables/figure_S6.pdf]

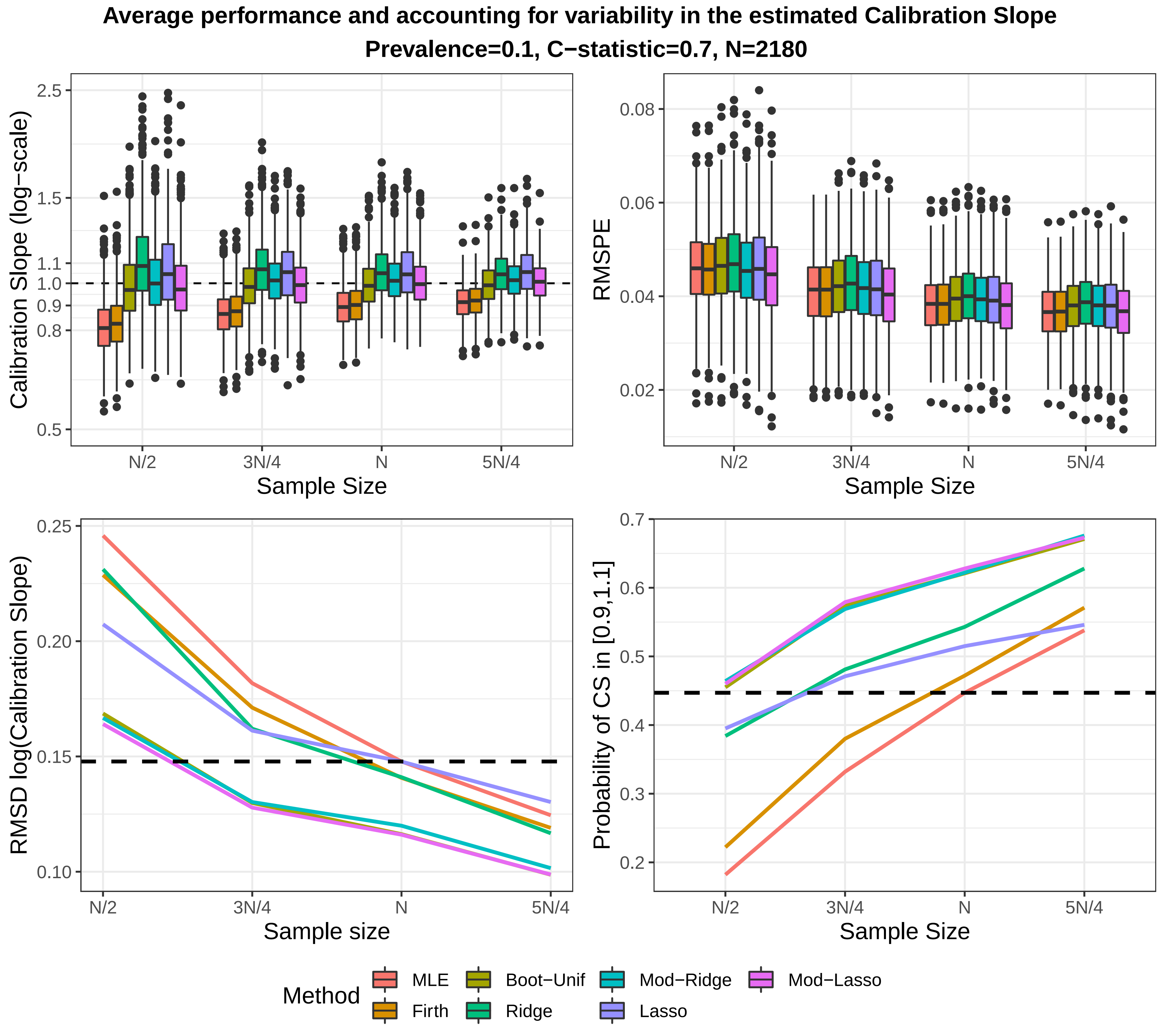
Average performance and accounting for variability in the estimated Calibration Slope
Prevalence=0.1, C−statistic=0.7, N=2180
Calibration Slope (log−scale)
2.5
1.5
1.1
1.0
0.9
0.8
0.5
N/2
3N/4
N
5N/4
Sample Size
RMSPE
0.08
0.06
0.04
0.02
N/2
3N/4
N
5N/4
Sample Size
RMSD log(Calibration Slope)
0.25
0.20
0.15
0.10
N/2
3N/4
N
5N/4
Sample size
Probability of CS in [0.9,1.1]
0.7
0.6
0.5
0.4
0.3
0.2
N/2
3N/4
N
5N/4
Sample Size
Method
MLE
Firth
Boot−Unif
Ridge
Mod−Ridge
Lasso
Mod−Lasso
