# Supplementary figures and images for: Penalized Regression Methods With Modified Cross‐Validation and Bootstrap Tuning Produce Better Prediction Models
Source: Biom J. 2024 Jun 24;66(5):e202300245. doi: 10.1002/bimj.202300245 (PMC12859537; doi:10.1002/bimj.202300245)

# Calibration Slope in individual datasets. Baseline method: MLE

Prevalence=0.5, C-statistic=0.7, N=680

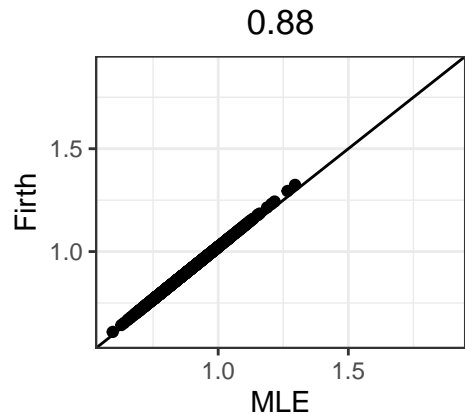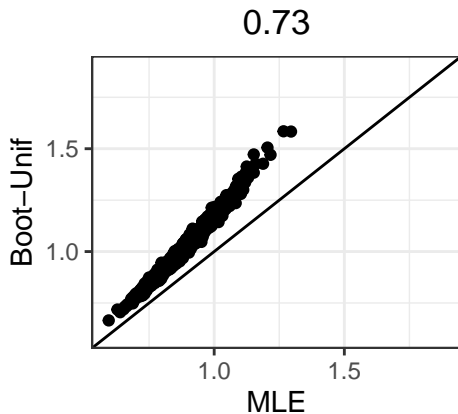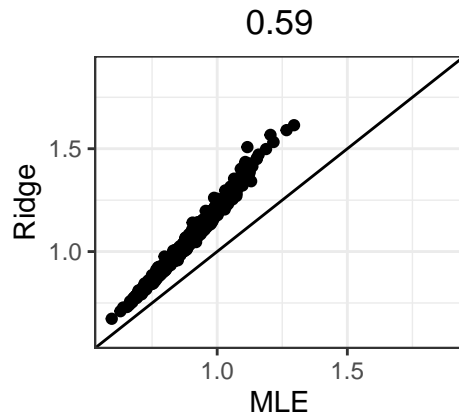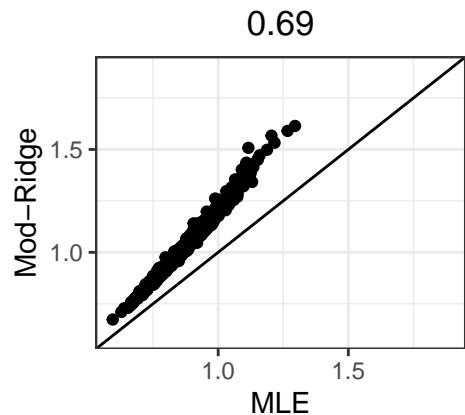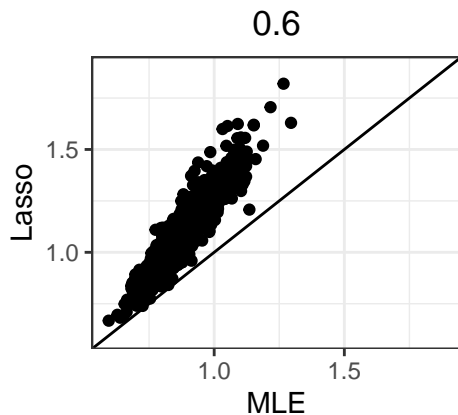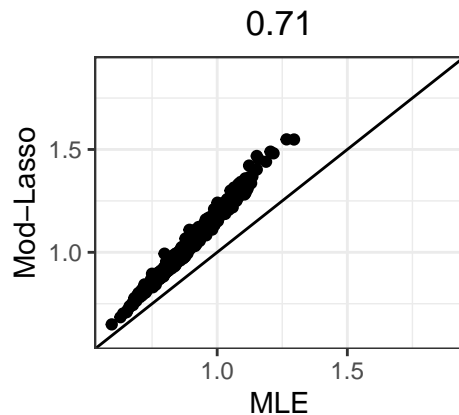

Supplement: Supplementary file 2 — Supporting Information [file BIMJ-66-e202300245-s002.zip › Supplementary_Material_2/figures_tables/figure_S2.pdf]

# C-statistic in individual datasets. Baseline method: MLE

Prevalence=0.5, C-statistic=0.7, N=680

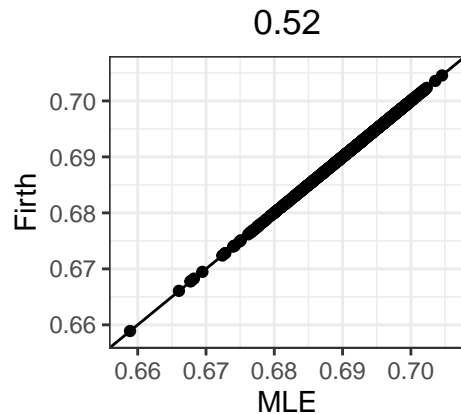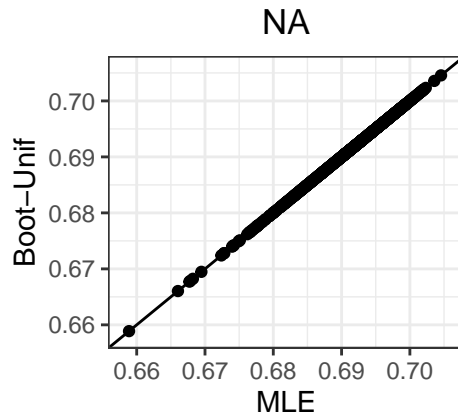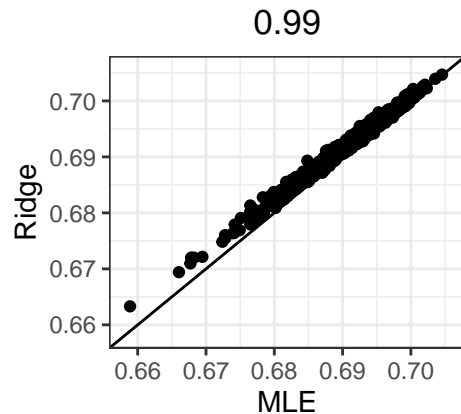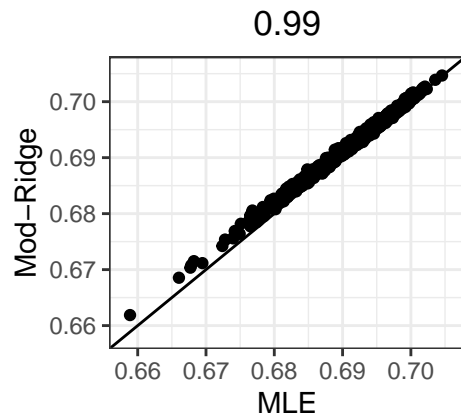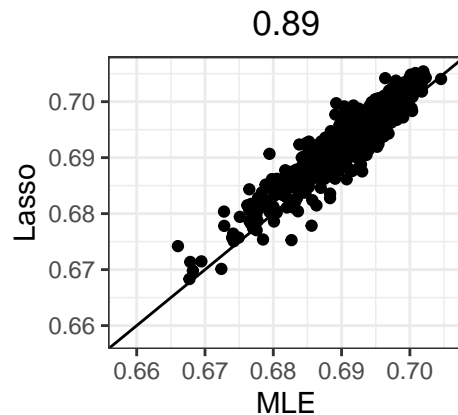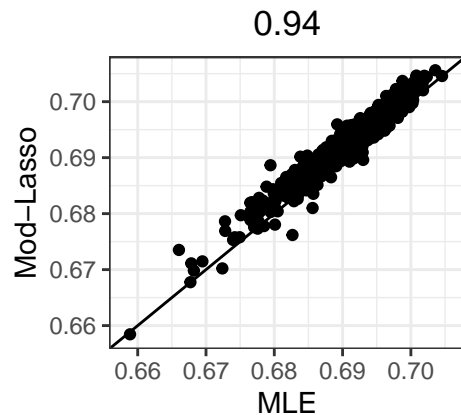

Supplement: Supplementary file 2 — Supporting Information [file BIMJ-66-e202300245-s002.zip › Supplementary_Material_2/figures_tables/figure_S3.pdf]

# RMSPE in individual datasets. Baseline method: MLE

Prevalence=0.5, C-statistic=0.7, N=680

0.87

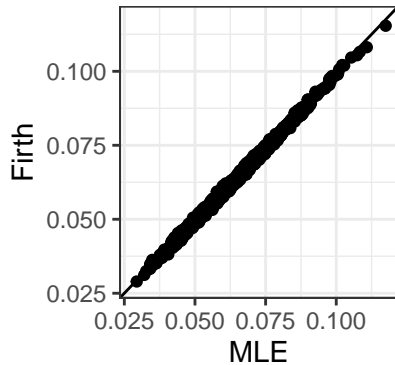

0.73

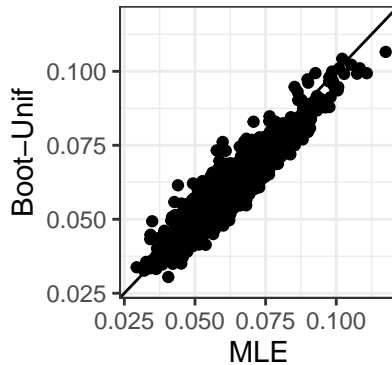

0.74

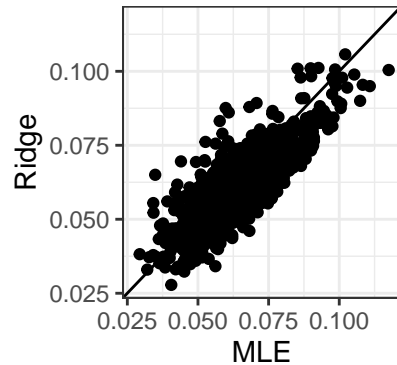

0.8

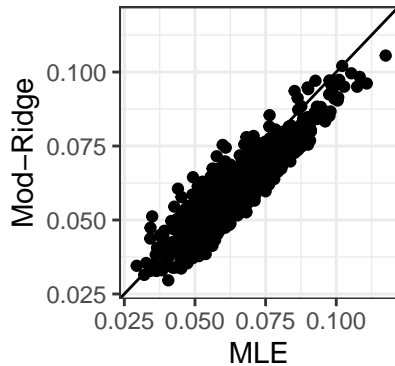

0.8

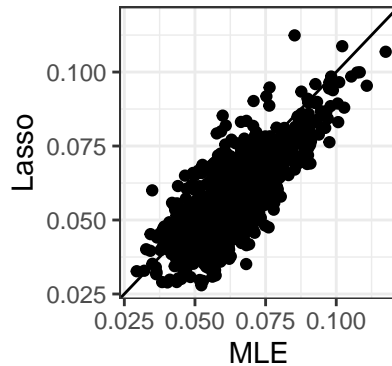

0.91

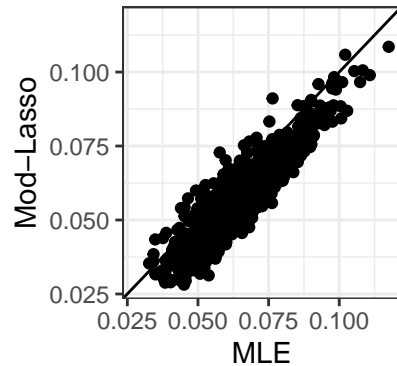

Supplement: Supplementary file 2 — Supporting Information [file BIMJ-66-e202300245-s002.zip › Supplementary_Material_2/figures_tables/figure_S4.pdf]

# Synthetic data application based on the Heart Valve Surgery data

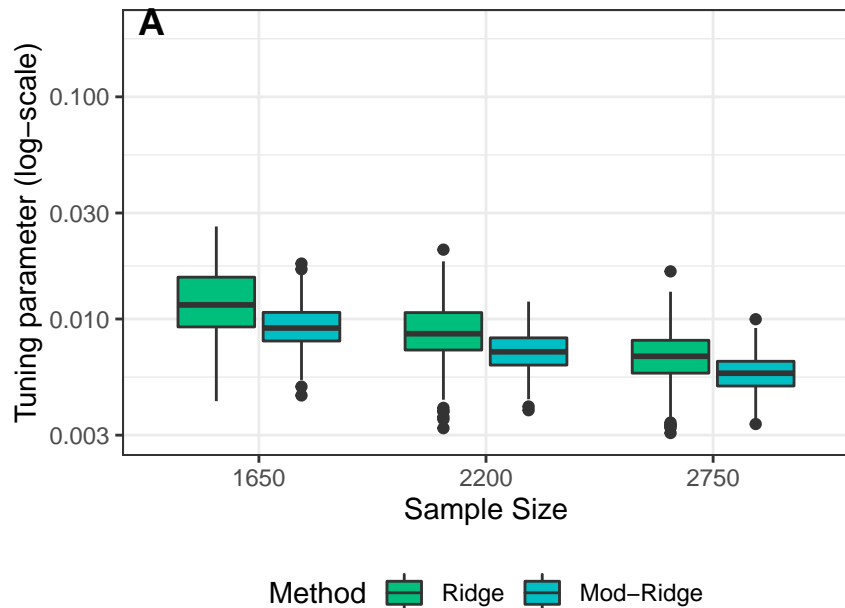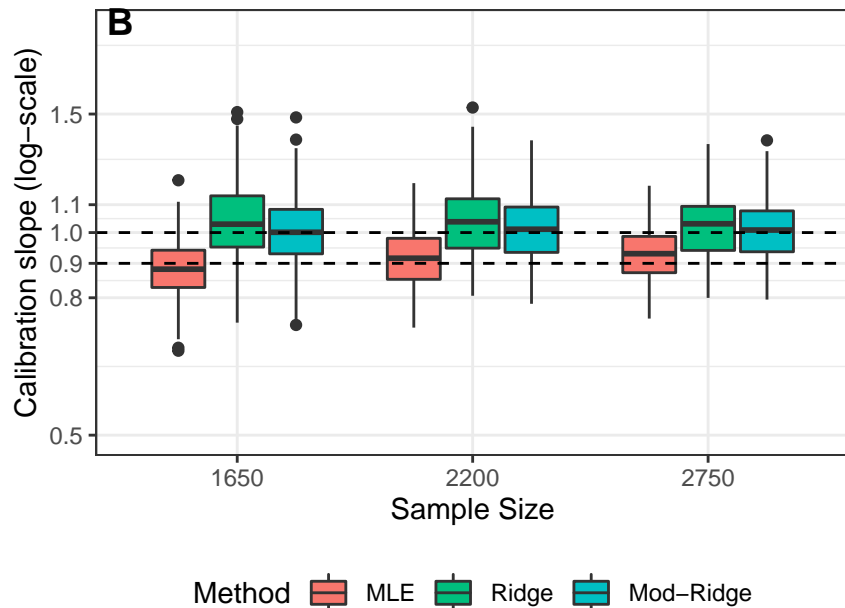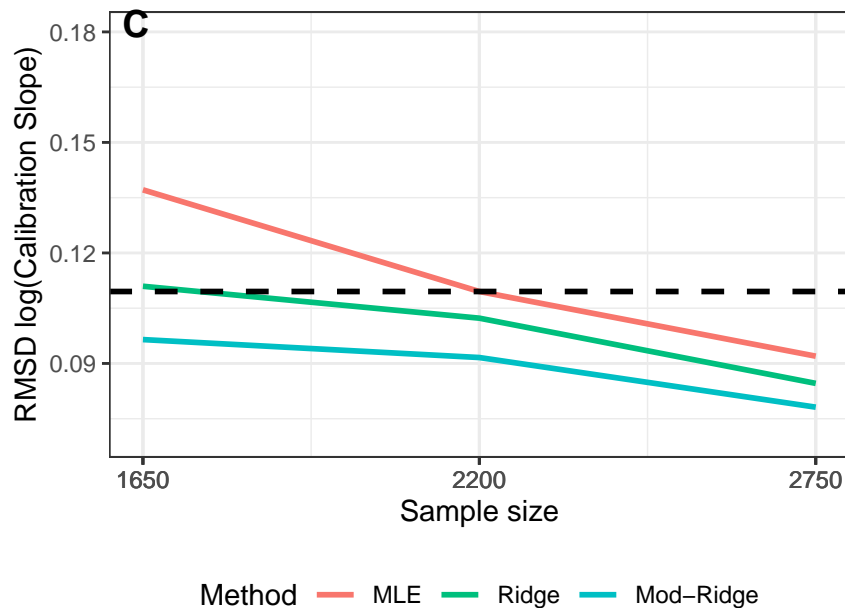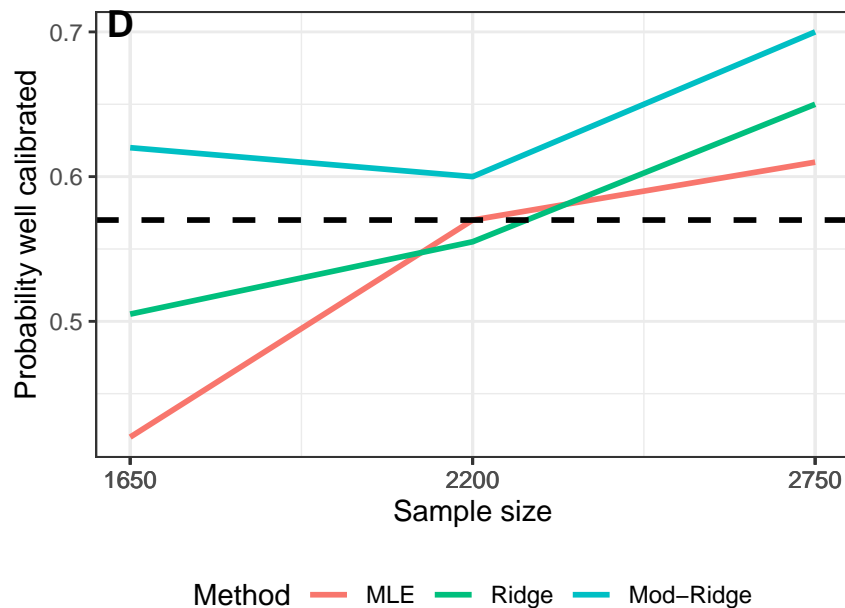

Supplement: Supplementary file 2 — Supporting Information [file BIMJ-66-e202300245-s002.zip › Supplementary_Material_2/figures_tables/figure_S9.pdf]
